# Computational identification of the selenocysteine tRNA (tRNA^Sec^) in genomes

**DOI:** 10.1371/journal.pcbi.1005383

**Published:** 2017-02-13

**Authors:** Didac Santesmasses, Marco Mariotti, Roderic Guigó

**Affiliations:** 1 Centre for Genomic Regulation (CRG), The Barcelona Institute for Science and Technology, Barcelona, Spain; 2 Universitat Pompeu Fabra (UPF), Barcelona, Spain; 3 Institut Hospital del Mar d’Investigacions Mèdiques (IMIM), Barcelona, Spain; 4 Division of Genetics, Department of Medicine, Brigham and Women’s Hospital, Harvard Medical School, Boston, Massachusetts, United States of America; University of Bristol, UNITED KINGDOM

## Abstract

Selenocysteine (Sec) is known as the 21st amino acid, a cysteine analogue with selenium replacing sulphur. Sec is inserted co-translationally in a small fraction of proteins called selenoproteins. In selenoprotein genes, the Sec specific tRNA (tRNA^Sec^) drives the recoding of highly specific UGA codons from stop signals to Sec. Although found in organisms from the three domains of life, Sec is not universal. Many species are completely devoid of selenoprotein genes and lack the ability to synthesize Sec. Since tRNA^Sec^ is a key component in selenoprotein biosynthesis, its efficient identification in genomes is instrumental to characterize the utilization of Sec across lineages. Available tRNA prediction methods fail to accurately predict tRNA^Sec^, due to its unusual structural fold. Here, we present Secmarker, a method based on manually curated covariance models capturing the specific tRNA^Sec^ structure in archaea, bacteria and eukaryotes. We exploited the non-universality of Sec to build a proper benchmark set for tRNA^Sec^ predictions, which is not possible for the predictions of other tRNAs. We show that Secmarker greatly improves the accuracy of previously existing methods constituting a valuable tool to identify tRNA^Sec^ genes, and to efficiently determine whether a genome contains selenoproteins. We used Secmarker to analyze a large set of fully sequenced genomes, and the results revealed new insights in the biology of tRNA^Sec^, led to the discovery of a novel bacterial selenoprotein family, and shed additional light on the phylogenetic distribution of selenoprotein containing genomes. Secmarker is freely accessible for download, or online analysis through a web server at http://secmarker.crg.cat.

## Author summary

Most proteins are made of twenty amino acids. However, there is a small group of proteins that incorporate a 21st amino acid, Selenocysteine (Sec). These proteins are called selenoproteins and are present in some, but not all, species from the three domains of life. Sec is inserted in selenoproteins in response to the UGA codon, normally a stop codon. A Sec specific tRNA (tRNA^Sec^), which only exists in the organisms that synthesize selenoproteins recognizes the UGA codon. tRNA^Sec^ is not only indispensable for Sec incorporation into selenoproteins, but also for Sec synthesis, since Sec is synthesized on its own tRNA. The structure of tRNA^Sec^ differs from that of canonical tRNAs, and general tRNA detection methods fail to accurately predict it. We developed Secmarker, a tRNA^Sec^ specific identification tool based on the characteristic structural features of the tRNA^Sec^. Our benchmark shows that Secmarker produces nearly flawless tRNA^Sec^ predictions. We used Secmarker to scan all currently available genome sequences. The analysis of the highly accurate predictions obtained revealed new insights into the biology of tRNA^Sec^.

## Introduction

Selenoproteins contain the non-universal amino acid selenocysteine (Sec), a selenium-containing cysteine analogue. Selenoproteins are present in the three domains of life [1–3]. An estimated ∼20% of the sequenced prokaryotic genomes encode selenoproteins [2, 4–6]. Among eukaryotes, selenoproteins are present across most metazoan lineages [7], although complete loss of selenoproteins has been reported in some insects [8–11] and nematodes [12]. Selenoproteins are missing in all fungi and land plant genomes [1]. Protist lineages show a scattered distribution of the Sec trait (i.e., the usage of Sec in selenoproteins) [6]. Although they constitute a very small fraction of the proteome of a given organism, selenoproteins cover important roles in antioxidant defense, redox regulation, thyroid hormone activation and others [13]. Many of them have been shown to be encoded by essential genes in mammals (e.g., [14–16]).

Selenoprotein biosynthesis requires a molecular system of *cis*- and *trans*-acting factors dedicated to the synthesis of Sec and to its insertion in the nascent polypeptide chain during translation [17]. Central to this system is the tRNA carrying Sec, tRNA^Sec^, which plays a key role in both Sec biosynthesis and insertion. Sec is unique for it is the only known amino acid in eukaryotes whose synthesis occurs on its tRNA, lacking its own tRNA synthetase. [18–21]. The tRNA^Sec^ is first misacylated with serine by seryl-tRNA synthetase (SerRS) to give Ser-tRNA^Sec^. In eukaryotes and archaea, serine is phosphorylated by O-phosphoseryl-tRNA kinase (PSTK), then the phosphoseryl moiety is converted to selenocysteine by Sec synthase (SecS, SepSecS). In bacteria, instead, Ser-tRNA^Sec^ is directly converted to Sec-tRNA^Sec^ by the bacterial Sec synthase (SelA). Both in prokaryotes and eukaryotes, the selenium donor for the synthesis of Sec is selenophosphate, which is, in turn, synthesized from selenide by selenophosphate synthetase (SPS/SelD). Sec is inserted in response to the UGA codon–normally a stop codon. During the translation of selenoprotein transcripts, the Sec-specific translation elongation factor (EF-Sec in eukaryotes and archaea, SelB in bacteria) brings Sec-tRNA^Sec^ to the ribosome [22] at the Sec encoding UGA codon upon recognition of a secondary structure in the mRNA, the Sec insertion sequence (SECIS), by the SECIS binding protein (SBP2 in eukaryotes, SelB in bacteria).

Due to the non canonical usage of the UGA codon, prediction of selenoprotein genes in genomes is a difficult task, ignored by virtually all widely used computational annotation pipelines. As a result, selenoprotein genes are usually mispredicted, being generally truncated at the 3’ (when UGA is assumed to be the stop codon) or 5’ end (when a AUG downstream of the Sec-encoding UGA is preferred as the site of translation initiation to an upstream AUG that would lead to an in-frame UGA codon). Methods dedicated specifically to the prediction of selenoprotein genes have been developed [23–25], but they still require some non-negligible human curation resources. The efficient identification of a genome marker for Sec utilization would be, in this regard, beneficial since it will help to allocate dedicated selenoprotein annotation resources only when needed. tRNA^Sec^ is one such marker. Unlike other components of the selenoprotein biosynthesis system, which participate also in other pathways and may thus be found in selenoproteinless genomes, tRNA^Sec^ is specific to selenoprotein-containing genomes [6, 8, 9, 12].

Prediction of tRNA^Sec^ is usually carried out with general purpose tRNA detection programs, namely tRNAscan-SE [26] and aragorn [27] (e.g., in [8, 28–30]). Even though the two programs have been thoroughly benchmarked for canonical tRNAs, they fail to accurately predict tRNA^Sec^ genes, often predicting them in selenoproteinless genomes, and failing to predict them in selenoprotein containing genomes [6].

Here we describe Secmarker, a computational pipeline to predict tRNA^Sec^ in genomes. Secmarker uses Infernal [31], and has two main components, first three manually curated covariance models (CMs), corresponding to tRNA^Sec^ in bacteria, archaea and eukaryotes. Second, a set of filters that reduce substantially the number of false positive produced by Infernal when using these methods. The non-universality of Sec utilization and the absence of tRNA^Sec^ in organisms without such trait allowed us to design a proper benchmark for tRNA^Sec^ predictions. Such a benchmark is impossible for the rest of tRNAs, all of which occur practically in all living organisms. Our results show that with the appropriate post-processing filters, Secmarker produces almost flawless tRNA^Sec^ predictions. Secmarker can quickly scan entire genomes. We ran it on about 10,000 eukaryotic and prokaryotic genomes currently available, and identified highly reliable tRNA^Sec^ gene candidates in 2,884 of them. Analysis of the results revealed a number of novel insights into the biology and evolution of tRNA^Sec^, including the identification of an unusual fold for the tRNA in bacteria, an eukaryotic intron-containing tRNA^Sec^, the discovery of a number of genomes containing multiple tRNA^Sec^ genes likely to be functional, and the tracing of the duplicated copy of human tRNA^Sec^, likely a pseudogene, to the root of hominids. Moreover, the analysis of the genomes with predicted tRNA^Sec^ genes led to the discovery of a novel bacterial selenoprotein family, allowed to refine the phylogenetic distribution of selenoprotein containing genomes within insects, and resulted in the identification of the first non-insect arthropod species lacking selenoproteins.

## Results

### Secmarker

Secmarker is a tRNA^Sec^ computational detection pipeline that runs Infernal [31] with three manually curated tRNA^Sec^ CMs for archaea, bacteria and eukaryotes. The program scans a nucleotide sequence with the three models using cmsearch from the Infernal package, filters the results, and assigns the cmsearch score to the predicted candidates (Materials and Methods).

**Fig 1:**
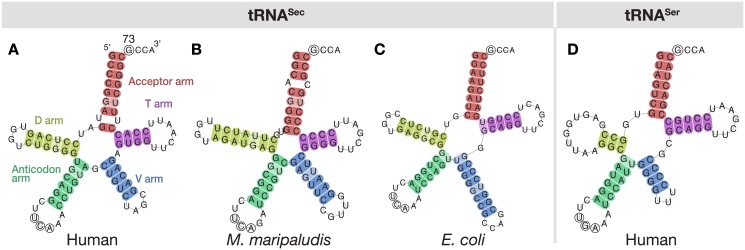
Secondary structure of tRNA^Sec^ and tRNA^Ser^. Cloverleaf models of tRNA^Sec^ (A–C) and of a canonical tRNA (tRNA^Ser^, D) in *Homo sapiens* (A and D, eukaryota), *Methanococcus maripaludis* (B, archaea) and *Escherichia coli* (C, bacteria). The acceptor arm, D arm, anticodon arm, variable arm and T arm are colored red, yellow, green, blue and purple, respectively. The anticodon triplet UCA (complementary to the UGA codon) is indicated with circled residues. The position 73, known as the discriminator base, is the fourth residue from the 3’ end, and is also circled. tRNA^Sec^ structures (A–C) were obtained with Secmarker. The tRNA^Ser^ structure (D) was obtained from tRNAdb 2009 [42]. The 3’ terminal CCA triplet is usually encoded in the genome in bacteria, while it is added post-transcriptionally in archaea and eukaryotes. The tRNA^Sec^ plots are examples of the graphical output of Secmarker.

The three models incorporate the structural features characteristic of tRNA^Sec^ in each of the three domains of life (Fig 1). The structure of tRNA^Sec^ comprises an aminoacyl acceptor arm (A-stem), a dihydrouridine arm (D-stem and D-loop), an anticodon arm (C-stem and C-loop, carrying the UCA anticodon complementary to UGA), a variable arm (V-stem and V-loop) and a TΨC arm (T-stem and T-loop). It is the longest tRNA, with 90–101 nucleotides, rather than the conventional ∼75 nucleotides in canonical tRNAs [32]. It has an unusual structure, different from the canonical 7/5 fold in other tRNAs (where 7 and 5 are the number of base pairs (bp) in the A and T stems, respectively). The tRNA^Sec^ adopts a 9/4 fold in eukaryotes [32, 33] and archaea [34], and a 8/5 fold in bacteria [35]. The acceptor and T arms form the AT-stem, which has 13 bp in tRNA^Sec^, compared to 12 bp in the usual 7/5 structure in other tRNAs. It has an exceptionally long variable arm, even longer than those of type-2 tRNAs (e.g., tRNA^Ser^) [35]. The D arm of tRNA^Sec^ has a long D-stem, with 6 bp in eukaryotes and bacteria, and 7 bp in archaea [36, 37], and a 4 bp D-loop, in contrast to the 3–4 bp D-stem and 7–12 nt D-loop in the canonical tRNAs [38]. Although SerRS recognizes both tRNA^Ser^ and tRNA^Sec^, the unique structure of tRNA^Sec^ is responsible for its specific interactions with PSTK [38, 39], SecS [33, 38] and EF-Sec in eukaryotes/archaea, and SelA [40] and SelB [41] in bacteria, discriminating tRNA^Sec^ from tRNA^Ser^.

The residue 73 in tRNAs, referred to as the discriminator base, is essential for aminoacylation by the corresponding aminoacyl-tRNA synthetase [43]. A guanine at this position (G73) is highly favored by SerRS [44]. Although tRNA^Ser^ possessing U73 have been observed in certain yeasts [45], tRNA^Sec^ carries a G73 in the three domains of life, which plays a critical role for the serylation by SerRS [19, 46, 47]. In fact, any mutation at this position prevents the aminoacylation of tRNA^Sec^ with serine [48]. Structure-based studies in both archaea and human showed that the residue G73 is also involved in latter steps of Sec formation. In archaea, during tRNA^Sec^ phosphorylation, G73 forms base-specific hydrogen bonds with conserved residues of PSTK [34]. Those residues are essential for PSTK activity in vitro and in vivo [34, 49]. In human, the interaction of SecS with the acceptor arm of tRNA^Sec^ involves base-specific hydrogen bonds between G73 and Arg398 [33]. Those interactions would be prevented by the substitution of G73 for any other nucleotide(A, C or U) [33]. In bacteria, the residues G1 and G73 in tRNA^Sec^ interact with the C-terminal region of SelA. Deletion of SelA residues 423 and 424, localized in the region that contacts G73, produces inactive enzymes [40]. The workflow of Secmarker includes the identification of the residue at position 73 in the tRNA^Sec^ candidates, but this residue is not included in the models or used to score candidates.

Secmarker is available for online analysis at http://secmarker.crg.cat, and it can also be downloaded and run locally. Secmarker requires a local installation of the Infernal package [31] and the ViennaRNA package [50]. The program analyzed ∼4MB/s in a single CPU (Intel(R) Xeon(R) CPU E5-2670 0 @ 2.60GHz) with 12GB of memory. See Materials and Methods for details.

### Benchmark of Secmarker

Unlike for the rest of tRNAs, it is possible to design a proper set for benchmarking predictions of tRNA^Sec^. This is because of the non-universality of Sec utilization trait and the absence of tRNA^Sec^ in organisms without such trait. Thus, tRNA^Sec^ predictions in selenoproteinless genomes are necessarily false positives, while lack of predictions in selenoprotein containing genomes correspond to false negatives. Analogous criteria cannot be employed for any other tRNA, since they are almost invariably present in the genomes of all organisms.

To design the benchmarking data set we used previous work in [6], where the presence of both selenoproteins and selenoprotein machinery factors was used to classify eukaryotic and bacterial genomes as either selenoprotein containing or selenoproteinless. This resulted in a set of 217 bacterial genomes (42 of which encode selenoproteins) and 212 eukaryotic genomes (105 of which encode selenoproteins). In addition, since archaea were not well represented in [6], we used Selenoprofiles [24] to scan 213 archaeal genomes for the presence of *selD* and *EF-Sec*, as well as selenoproteins. After manual curation of the results, we identified 14 genomes (6%) that use Sec. In total, therefore, our benchmark set included 642 sequenced genomes, of which 161 (25%) encoded selenoproteins (positive set) and 481 (75%) did not (negative set).

To evaluate the accuracy of tRNA^Sec^ predictions at the genome level, we computed sensitivity, as the fraction of genomes from the positive set in which at least one tRNA^Sec^ gene was predicted, and specificity as the fraction of genomes in the negative set in which no tRNA^Sec^ was predicted. This benchmark, however, is not perfect at the individual prediction level; since the correct tRNA^Sec^ loci are generally not known, true positives are overestimated and false positives underestimated, leading to overestimations of both sensitivity and specificity. Indeed the prediction of a wrong tRNA^Sec^ locus in a selenoprotein encoding genome will be considered in our approach to be a true positive, when actually it is a false positive. This is partially alleviated by the fact that selenoprotein encoding genomes possess normally a single tRNA^Sec^ locus [13]. Thus, the number of tRNA^Sec^ predicted genes in a given genome is also an indirect measure of specificity.

We ran aragorn (v1.2) [27], tRNAscan-SE (v1.23) [26] and Secmarker in our benchmark data set. Results are reported in Table 1. Using [6] as reference for selenoprotein containing genomes, Secmarker achieved globally both higher sensitivity than aragorn and tRNAscan-SE (99% vs 96% and 68%, respectively) and higher specificity (99% vs 83% and 89%, respectively), as well as within each domain/taxa considered (Table 1). Moreover, Secmarker predicted much fewer tRNA^Sec^ candidates (1.7 on average in selenoprotein containing genomes) than tRNAscan-SE (47.1) and aragorn (20.0). Since most multiple tRNA^Sec^ predictions in a given genome are likely to be false positives (see below), our measures of sensitivity and specificity actually underestimate the gap in performance between Secmarker and the other programs.

**Table 1:** Performance statistics of tRNA^Sec^ prediction for the three programs tested.

	Sets		Secmarker				tRNAscan-SE				aragorn				RF01852			
Lineage	+	-	sn	sp	N+	N-	sn	sp	N+	N-	sn	sp	N+	N-	sn	sp	N+	N-
*Full set*	161	481	99.4	99.4	1.7	0.0	67.5	88.6	47.1	0.2	96.2	83.0	20.0	0.4	98.8	90.2	2.4	0.1
*Metazoa*	55	15	100.0	100.0	2.3	0.0	92.7	73.3	135.7	1.1	100.0	66.7	55.2	0.4	100.0	86.7	4.1	0.2
*Fungi*	0	42	NA	100.0	NA	0.0	NA	45.2	NA	0.8	NA	38.1	NA	1.7	NA	100.0	NA	0.0
*Viridiplantae*	5	31	100.0	100.0	1.2	0.0	60.0	71.0	1.0	1.0	80.0	41.9	1.8	2.2	100.0	74.2	1.4	0.4
*Other euk*.	45	19	97.8	94.7	1.6	0.1	22.2	73.7	0.6	1.2	88.9	68.4	2.0	0.6	97.8	88.9	1.8	0.2
*Bacteria*	42	175	100.0	98.9	1.1	0.0	85.7	92.0	0.9	0.1	100.0	87.4	1.1	0.1	100.0	82.9	1.4	0.2
*Archaea*	14	199	100.0	100.0	1.1	0.0	57.1	100.0	0.6	0.0	92.9	97.5	1.1	0.0	92.9	97.5	0.9	0.0

Number of genomes in the positive (+) and negative (-) set, sensitivity (sn) and specificity (sp), and average number of predictions per genome in the positive (N+) negative set (N-).

In addition to tRNAscan-SE and aragorn, we also used RF01852 (Rfam tRNA-Sec) with Infernal 1.1 [31]. RF01852 achieved similar sensitivity than Secmarker, although the specificity was lower in prokaryotes and eukaryotes (Table 1 and S1 Text). It predicted 68% more tRNA-Sec genes than Secmarker, very likely to be false positives. In addition to having a superior performance, Secmarker has the advantage of identifying the domain to which the tRNA^Sec^ encoding genome belongs (bacteria, archaea or eukaryota). This can be particularly useful in the analysis of metagenomic data, where generally there is no previous knowledge of the sequenced genomes.

**Fig 2:**
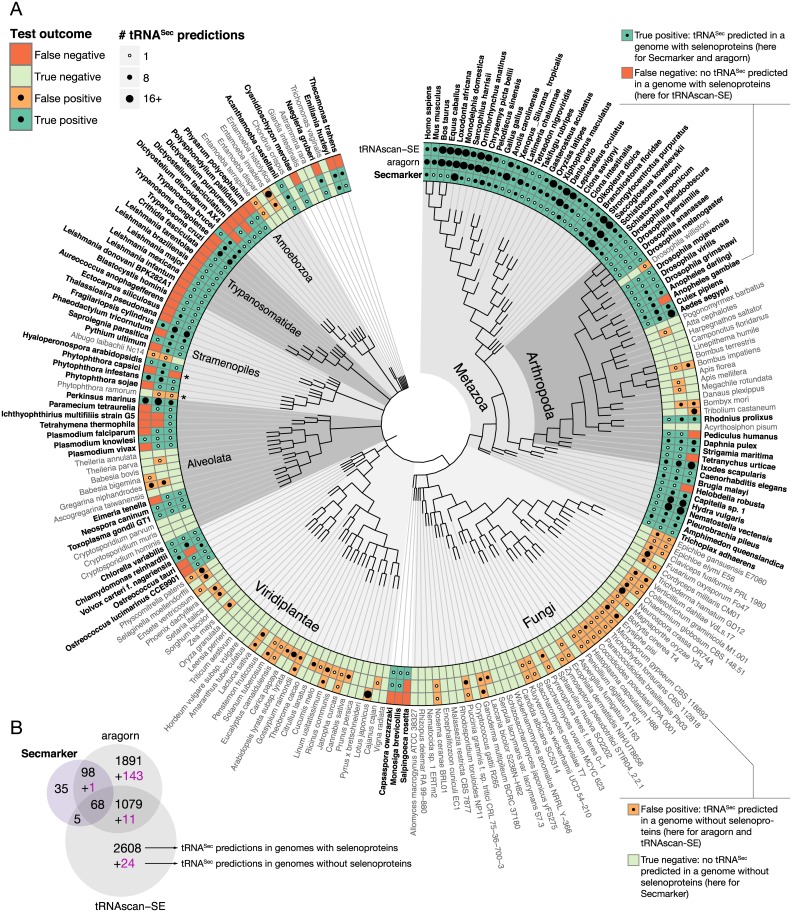
tRNA^Sec^ predictions in eukaryotic genomes. (A) Phylogenetic tree of the eukaryotic genomes used in the benchmark set. Sec-containing species are drawn in bold font. The tRNA^Sec^ predictions are indicated with dots. The size of each dot is proportional to the number of predictions. Open dots indicate a single prediction. The color of the cells indicate the outcome of the test, for each program. Species marked with a star (*) are discussed in the Results section and/or S1 Text. The approximate species phylogeny was obtained from the NCBI Taxonomy database (http://www.ncbi.nlm.nih.gov/taxonomy). Figure produced using our R package ggsunburst, available at http://genome.crg.es/∼dsantesmasses/ggsunburst. (B) Venn diagram showing the overlap between the tRNA^Sec^ genes predicted by the three programs. Numbers in black correspond to predictions in Sec-containing genomes. Purple numbers correspond to predictions in Sec-devoid genomes.

**Fig 3:**
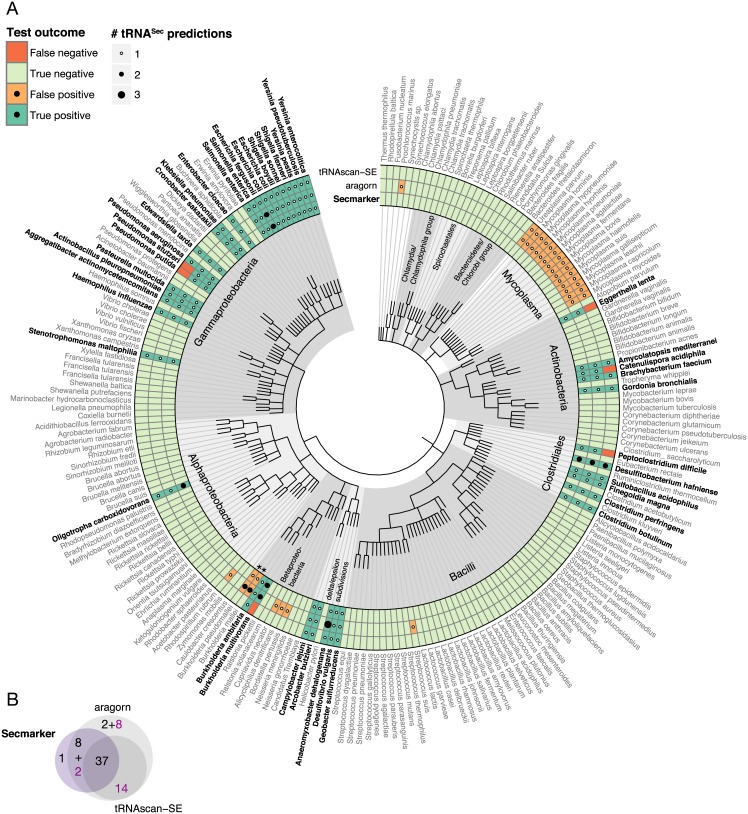
tRNA^Sec^ predictions in bacterial genomes. (A) Phylogenetic tree of the bacterial genomes used in the benchmark set. Sec-containing species are drawn in bold font. Genome names were cut down to species level (not including the strain) for visualization purposes. The complete names including strain identifiers are provided in S1 Table. Species marked with a star (*) are discussed in the Results section and/or S1 Text. (B) Venn diagram showing the overlap between the tRNA^Sec^ genes predicted by the three programs. See Fig 2 caption for details.

**Fig 4:**
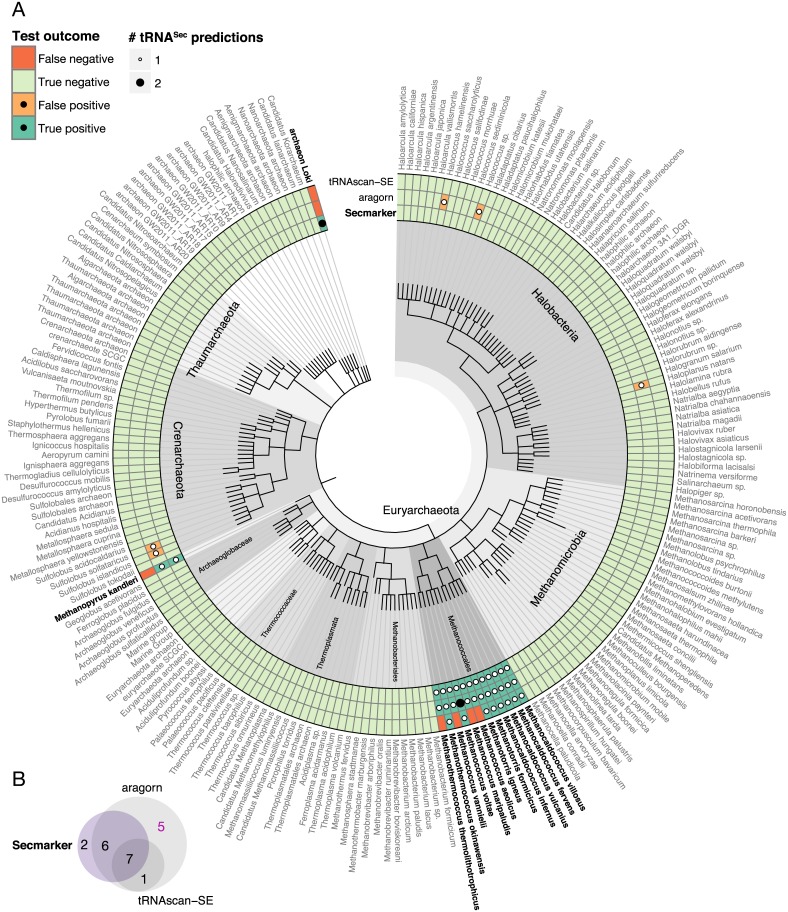
tRNA^Sec^ predictions in archaea genomes. (A) Phylogenetic tree of the archaeal genomes used in the benchmark set. Sec-containing species are drawn in bold font. Genome names were cut down to species level (not including the strain) for visualization purposes. The complete names including strain identifiers are provided in S1 Table. (B) Venn diagram showing the overlap between the tRNA^Sec^ genes predicted by the three programs. See Fig 2 caption for details.

Figs 2, 3 and 4 summarize the tRNA^Sec^ predictions obtained by the three programs in eukaryotes, bacteria and archaea (see S1 Text for details). At the genome level Secmarker produced only one apparently false negative prediction, and three apparent false postive predictions. Secmarker failed to predict tRNA^Sec^ candidates in the genome of the selenoprotein containing protist *Phytophthora capsici* (Fig 2). Using Secmarker, however, on the raw sequence reads available for this genome, we identified a full length tRNA^Sec^ gene (section 5 in S1 Text). Secmarker, thus, failed to predict it because the gene sequence is missing from the genome assembly analyzed here.

On the other hand, Secmarker predicted tRNA^Sec^ genes in three genomes annotated in [6] as lacking selenoproteins: the eukaryote *Phytophthora ramorum*, and two bacteria from the genus *Burkholderia*. In all these cases, analysis of more recent assemblies indicated that these genomes encode selenoproteins, since we identified key genes for selenoprotein biosynthesis as well as selenoproteins themselves (S1 Text). Secmarker therefore correctly predicted tRNA^Sec^ genes in these genomes. Evaluated at the genome level, therefore, Secmarker produces flawless predictions, and these are a perfect marker for selenoprotein containing genomes.

While there was good overall overlap between Secmarker, aragorn and tRNAscan-SE predictions in bacteria (Fig 3) and archaea (Fig 4), there were large discrepancies in eukaryotes (Fig 2). Both aragorn and tRNAscan-SE produced numerous false positive predictions in fungi and land plants, both known to lack selenoproteins [1]. On the other hand, there was substantial overlap between gene predictions from aragorn and tRNAscan-SE in genomes with the Sec trait, that were not predicted by Secmarker (1,079 genes, Fig 2B). Even though these predictions were obtained from selenoprotein encoding genomes, we considered them very unlikely to be correct, because nearly all of them (99%) were predicted in just four genomes, those of *Bos taurus* (487), *Ornithorhynchus anatinus* (478), *Loxodonta africana* (50) and *Danio rerio* (21), and selenoprotein containing eukaryotic genomes are known to normally encode only one or very few tRNA^Sec^ genes (see below).

### tRNA^Sec^ across genomes

In addition to the benchmark set, we ran Secmarker on the genome sequences available for 9,780 organisms. We initially predicted 3,341 tRNA^Sec^ genes in 2,899 genomes (Table 2). The analysis of the Secmarker results revealed a number of insights on the biology, structure and evolution of tRNA^Sec^.

**Table 2:** Total number of genomes analyzed and tRNA^Sec^ predictions by Secmarker.

Lineage	Genomes	Genomes with tRNA^Sec^	tRNA^Sec^ genes
Root	9780	2899	3341
*Bacteria*	8233	2316	2362
*Eukaryota*	1049	562	957
*Archaea*	498	21	22

#### The discriminator base in tRNA^Sec^

**Fig 5:**
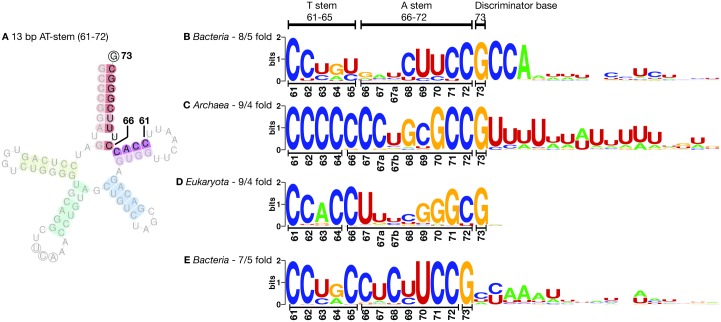
The discriminator base G73 in tRNA^Sec^. Sequence logos [53] of the 3’ end of tRNA^Sec^ candidates from the three domains of life. The subsequences include the AT-stem, starting at position 61 of tRNA (numbering based on [35]) and extends further into the 3’ region of the gene. The residue in position 73 (the discriminator base) shows a strongly conserved G (guanine). (A) 9/4 fold tRNA cloverleaf structure indicating the 13 base pairs acceptor plus T-arm sequence used in the logos. (B) Bacteria, 2316 sequences; (C) archaea, 20 sequences; (D) eukaryota, 562 sequences; (E) bacterial 7/5 fold tRNA^Sec^ candidates, including 47 sequences with a shorter 12 bp AT-stem. tRNAs have a poly-T motif in the 3’ region as the transcription termination signal [54], here only visible in archaea because of the low number of sequences. Only the top scoring candidate in each genome were used to generate the logos.

To assess the quality of the predictions at the individual level, we investigated the nucleotide present at the residue 73 of tRNA^Sec^ candidates in the extended set of genomes. Across all analyzed genomes, the great majority of the tRNA^Sec^ candidates predicted by Secmarker, 3,162 out of 3,341 (94.6%) contained the canonical guanine at position 73 (G73), as reflected in the multiple alignment of all the highest scoring Secmarker predicition in each genomes (Fig 5). In bacteria, following the G73, we observed a conserved CCA triplet, the universal 3’ end of mature tRNAs [51]. The triplet is generally encoded in the genome in bacteria (93% of the tRNA^Sec^ genes), but not in archaea (5%) and eukaryotes (3%), as previously observed for canonical tRNAs [52].

There were 178 tRNA^Sec^ candidates in 125 genomes with a nucleotide different than a G in position 73. In 61 genomes, the non G73 candidate was either the sole prediction or the top scoring one. Nine such predictions were in vertebrate genomes (*Monodelphis domestica*, *Haliaeetus albicilla*, *Opisthocomus hoazin*, *Fulmarus glacialis*, *Egretta garzetta*, *Tinamus guttatus*, *Cariama cristata*, *Struthio camelus* and *Phalacrocorax carbo*). The remaining 52 were all in bacteria, and the analysis of the sequences led us to identify an unusual tRNA^Sec^ structure (see next section).

#### Unusual 12 base pairs AT-stem in tRNA^Sec^

The total length of the tRNA^Sec^ acceptor stem plus T-stem is 13 bp (8+5 in bacteria [35] or 9+4 in archaea and eukaryotes [33]). Deviations from the bacterial 8+5 structure have been recently reported in [55] and [56]. The former described tRNA^Sec^ genes from *Epsilonproteobacteria* with 12 bp AT-stem plus one bulged nucleotide, and the latter described the *Cloacimonetes* type tRNA^Sec^, which has 12 bp (7+5) and lacks one nucleotide in the linker region between the acceptor stem and D-stem.

Among the 52 non G73 bacterial tRNA^Sec^ identified in this study, detailed analysis revealed that 47 had a 12 bp AT-stem. Similar to the *Cloacimonetes* type [56], they had a 7 bp acceptor stem (7 residues between the T-stem and the discriminator base G73). Secmarker initially failed to correctly identify the G73 residue since it relies on the assumption of a 13 bp AT-stem, but their structural alignment actually revealed a conserved residue G73, and the CCA tail in some of them (S1 Fig). These tRNA^Sec^ sequences were found in several genomes from *Gammaproteobacteria*, *Clostridiales*, *Spirochaetes*, in two species of *Alphaproteobacteria*, and in *Rubrobacter xylanophilus DSM 9941* (*Actinobacteria*) and *Dehalogenimonas lykanthroporepellens BL-DC-9* (*Dehalococcoidetes*), although not all tRNA^Sec^ genes in these lineages exhibited the 7/5 fold. Most of these tRNA^Sec^ had a bulged nucleotide in the acceptor stem, based on the inferred secondary structure (S1 and S2 Figs). The bulged nucleotide was observed in different positions (S2 Fig; columns A, B and C). Several tRNA^Sec^ from *Alphaproteobacteria* and *Gammaproteobacteria* had an extra nucleotide in the linker region between the acceptor stem and D-stem (position 7a) while lacking the bulged nucleotide in the acceptor stem (S2 Fig; column D). *R. xylanophilus DSM 9941* tRNA^Sec^ lacked one nucleotide in the linker region between the acceptor stem and D-stem (S1 Fig). A common feature amongst most of the 12 bp AT-stem tRNA^Sec^ was a bulged nucleotide in the anticodon stem (position 43a). Also, specific to *Clostridiales*, a bulged nucleotide in the D-stem (position 13a) was observed. The tRNA residues numbering was based in [35]. The remaining five non G73 tRNA^Sec^ bacterial top scoring candidates are shown in S1 Text.

In the genomes where these unusual tRNA^Sec^ candidates were identified, we also predicted Sec-containing genes and the genes encoding the protein factors of the Sec machinery: *selA*, *selB* and *selD*. With few exceptions, tRNA^Sec^ (*selC*) was found very close to *selA* and *selB* genes, forming a *selABC* operon (S2 Fig). Some of the genomes had two non-identical copies of tRNA^Sec^, which were located adjacent to each other in the same operon, in the case of four *Clostridiales* genomes, or in two different complete operons, in the case of *Photobacterium profundum 3TCK* (S2 Fig). Despite their unusual structure, these observations suggest that these tRNA^Sec^ are indeed involved in Sec synthesis and incorporation.

#### Multiple tRNA^Sec^ predictions, and tRNA^Sec^ pseudogenization

After taking into account the 7/5 bacterial fold, only 14 tRNA^Sec^ candidates among the 2,898 that were the sole or the top ranking prediction lacked a G at the position 73: the nine in vertebrates and the five in bacteria mentioned above. As these candidates had one or more disrupted pairs in their inferred secondary structure, they are most likely not functional–the functional tRNA^Sec^ genes likely missing from the genome assemblies of these species–and they should, therefore, be considered Secmarker false positives. Furthermore, among the genomes (193) in which Secmarker predicted multiple tRNA^Sec^ genes, there were 117 non G73 predictions. They scored much lower than the G73 predictions, irrespective of whether they were or not the top ranking prediction (S3 Fig).

From the analysis above, we conclude that G at position 73 is essential for tRNA^Sec^ function. In total, Secmarker predicted 3213 G73 tRNA^Sec^ genes in 2884 genomes.

**Fig 6:**
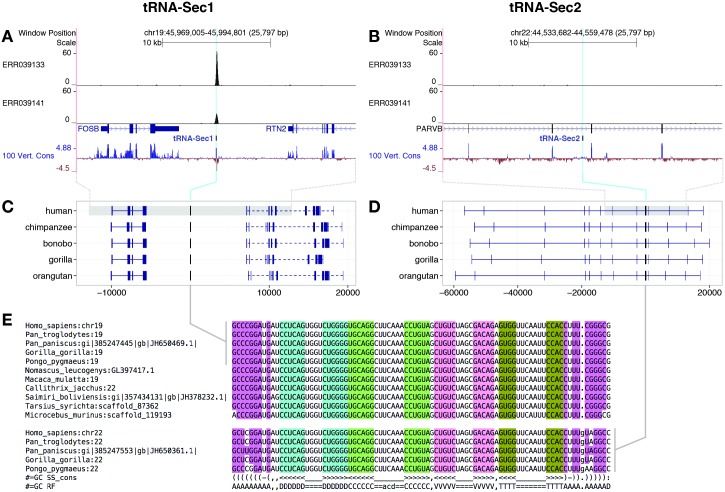
Duplication of tRNA^Sec^ in hominids. Pol III binding in human *tRNA^Sec^1* (A) and *tRNA^Sec^2* (B) by Chip-seq (tracks ERR039133 and ERR039141, see Materials and Methods). Conserved syntenic genes surrounding *tRNA^Sec^1* (C) and *tRNA^Sec^2* (D) in the genome of five hominids. *tRNA^Sec^1* is flanked by the genes *FOSB* and *RTN2*, and *tRNA^Sec^2* is located within an intron of *PARVB*. (E) Structural alignment of *tRNA^Sec^1* in eleven primates (top) and *tRNA^Sec^2*, only found in hominids (bottom). Panels A and B were produced with the UCSC genome browser [60] on the human hg19 assembly. “100 Vert. Cons” track corresponds to sequence conservation across 100 vertebrates. Protein coding annotations in panels C and D were obtained with Selenoprofiles [24]. Sequences in panel E were obtained with Secmarker, aligned using Infernal (cmalign program) [31], and visualized with RALEE [61]. RALEE highlights the nucleotides that are paired according to the consensus secondary structure at the bottom of the alignment, and that also respect the standard pairing rules. The rightmost column in the alignment corresponds to the discriminator base.

Non G73 Secmarker predictions could partially reflect pseudogenization events. tRNA^Sec^ pseudogenes have been previously described in rabbits, Chinese hamsters and humans [54, 57]. Here we investigated in detail the origin and evolutionary fate of the human tRNA^Sec^ pseudogene. The human tRNA^Sec^ was first identified as an opal suppressor gene [54] located on the chromosome 19 [58]. Along with the gene, known as *TRNAU1* (here named *tRNA^Sec^1*), a second copy was also identified [54] on chromosome 22 [58]. The second human tRNA^Sec^, known as *TRNAU2* (here named *tRNA^Sec^2*) presents features that suggest pseudogenization [54]: it has a cytosine discriminator base, and several pairs in the acceptor stem are disrupted. Secmarker identified the two genes in their expected genomic locations in the genomes of hominids, but only *tRNA^Sec^1* in the genome of other primates (Fig 6E). The homology between the two tRNA^Sec^ is limited to the sequence of the mature tRNA, and several repetitive elements belonging to the ALU family are present surrounding *tRNA^Sec^2* [54]. These observations suggest that *tRNA^Sec^2* originated by retrotransposition of *tRNA^Sec^1* in the lineage of Apes, after the split with *Nomascus* and before the split of *Pongo*. As in human, *tRNA^Sec^2* has a discriminator base cytosine in all analyzed hominids, whereas *tRNA^Sec^1* has always guanine (Fig 6E). We searched for evidences of transcription of human *tRNA^Sec^1* and *tRNA^Sec^2*. tRNA genes are transcribed by RNA polymerase (Pol) III [59]. Pol III occupancy at tRNA loci, and importantly to their unique flanking regions, has been used to measure tRNA genes usage [59]. We processed Pol III Chip-seq data performed on human liver samples from [59], and we found evidence of Pol III bound to *tRNA^Sec^1* (Fig 6A), but not to *tRNA^Sec^2* (Fig 6B), in two different samples. From all these observations, it would seem that *tRNA^Sec^2* was “dead on arrival” after its origin by retrotransposition.

In general, it is assumed that tRNA^Sec^ occurs as a single copy functional gene [13]. Consistently, tRNA^Sec^ knockout mice showed an early embryonic lethal phenotype [62]. So far, only in one genome, that of zebrafish, two tRNA^Sec^ genes have been reported [63], which are completely identical in sequence. However, even ignoring the non G73 predictions, we still found 151 genomes with two or more G73 tRNA^Sec^ genes predicted by Secmarker (478 predictions in total). The score of the additional G73 copies was higher than the non G73 copies (S3 Fig; note that the residue at position 73 does not contribute to the Secmarker score of the predictions). This suggests that some of these could be functional. Analysis of these predictions, however, revealed that many of them are 100% identical in sequence, even when including the 100 bp flanking regions, suggesting artefacts in genome assembling. After discarding identical predictions, 376 candidates in 124 genomes remained. Detailed analysis on the 252 G73 copies (not including the top scoring candidate in these genomes) revealed that 145 predictions (80 genomes) had mutations, when compared to the top scoring one, that would disrupt the tRNA structure, and they are thus likely to be Secmarker false positives. Among the remaining predictions, one is likely to be a contaminant (a protist tRNA in a bird genome), and 69 predictions (46 genomes) did not have mutations or the mutations did not affect the pairing potential of the sequence. Interestingly, 27 predictions (21 genomes) had compensatory mutations when aligned to the top scoring candidate (Table 3). Many of these are likely to be“bona fide” tRNA^Sec^ genes. While some genomes with multiple tRNA^Sec^ genes have many selenoproteins, others have very few. Eighteen genomes (eight bacterial and ten eukaryotes) had two tRNA^Sec^ genes (i.e., the top scoring one and an additional copy with compensating mutations), and three eukaryotes had four tRNA^Sec^ genes. In the genomes of the common spider *Parasteatoda tepidariorum* and the lancelet *Branchiostoma floridae*, the compensating mutations in the duplicated copies were identical, suggesting that they occurred in the first duplicated copy before subsequent duplications. Strikingly, the genome of the diatom *Fragilariopsis cylindrus* had eleven non-identical predictions (taking into account the 100 nt flanking sequence). Two of them had a mutation that would disrupt the tRNA structure. Among the remaining nine, three showed compensatory mutations when compared to the top scoring one. Two of the duplicated copies showed the same compensatory mutations (S4 Fig).

**Table 3:** Species with multiple tRNA^Sec^ candidates.

	Species	tRNA^Sec^	selenoproteins
Eukaryotes	*Fragilariopsis cylindrus* (Diatom)	4	36
	*Branchiostoma floridae* (Lancelet)	4	25
	*Parasteatoda tepidariorum* (Common house spider)	4	12
	*Lingula anatina* (Brachiopod)	2	34
	*Latimeria chalumnae* (Coelacanth)	2	27
	*Lepisosteus oculatus* (Bony fish)	2	27
	*Lepeophtheirus salmonis* (Crustacean)	2	17
	*Daphnia pulex* (Crustacean)	2	16
	*Centruroides exilicauda* (Scorpion)	2	13
	*Volvox carteri f. nagariensis* (Green algae)	2	8
	*Machilis hrabei* (Insect)	2	7
	*Gyrodactylus salaris* (Flatworm)	2	6
	*Belgica antarctica* (Insect)	2	2
Bacteria	*Desulfosporosinus orientis DSM 765* (Clostridia)	2	13
	*Desulfitobacterium dehalogenans ATCC 51507* (Clostridia)	2	5
	*Halanaerobium praevalens DSM 2228* (Clostridia)	2	5
	*Desulfitobacterium hafniense DP7* (Clostridia)	2	4
	*Desulfitobacterium hafniense Y51* (Clostridia)	2	4
	*Shigella flexneri 1235–66* (Enterobacteria)	2	4
	*Clostridiales bacterium 1_7_47FAA* (Clostridia)	2	3
	*Clostridium citroniae WAL-17108* (Clostridia)	2	2

Number of tRNA^Sec^ candidates (including the top scoring one plus those that exhibit compensatory mutations when aligned to the top scoring one) and number of predicted selenoprotein genes.

We did not find any correlation between the number of tRNA^Sec^ in genomes with multiple candidates and the number of selenoproteins.

#### A novel selenoprotein family

Aiming to gain insights into the evolution of the Sec encoding trait, we searched for the rest of the Sec machinery and selenoprotein genes in all genomes using Selenoprofiles [24]. Our results in prokaryotes were overall consistent with previous reports. Across genomes, the presence of tRNA^Sec^ correlated well with the presence of selenoproteins and selenoprotein machinery. The most common exceptions were genomes with a *SelD* gene (selenophosphate synthetase) but no tRNA^Sec^ or selenoproteins, consistent with *SelD* supporting Se utilization traits other than Sec [5, 6]. Our search also identified four selenoprotein genes in two archaeal assemblies (the *Euryarchaeota* strains SCGC AAA261-G15 and SCGC AAA288-E19) without predicted tRNA^Sec^; however all four genes had a bacterial SECIS (identified using [64]), thus very likely reflecting bacterial contamination in the assemblies.

**Fig 7:**
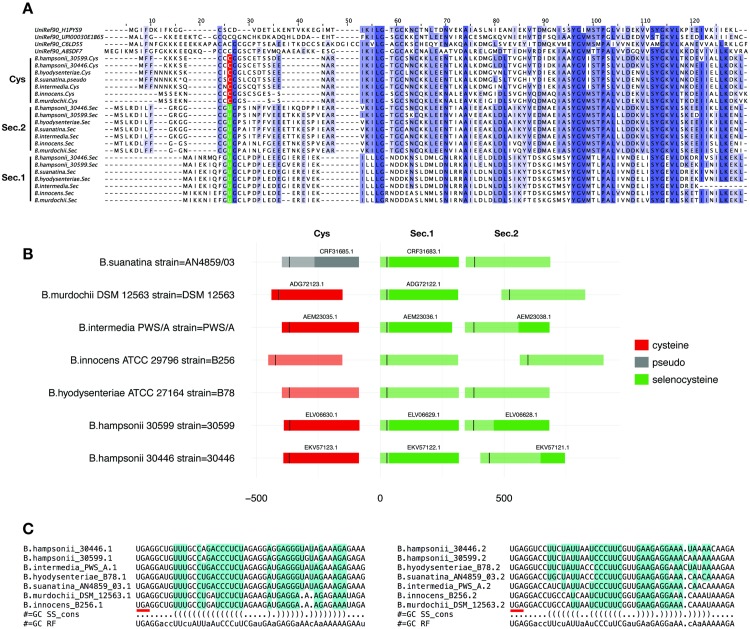
“Redox-active disulfide protein 2” selenoproteins in *Brachyspira*. (A) Multiple sequence alignment containing amino acid sequences obtained from UniRef90 (top four) and from *Brachyspira* genomes using Selenoprofiles [24]. In the *Brachyspira* sequences, the Sec position (column 26) is coloured according to the codon found in the genome: Cys in red; and Sec in green. The thioredoxin domain spans from column 53 to the C-terminus. (B) Genomic arrangement of the three “Redox-active disulfide protein 2” genes, all of them found in a gene cluster in each of the *Brachyspira* genomes (rows). The genes are coloured according to the codon in the Sec position (marked in black), following the same colouring scheme as panel A. Selenoproteins were either missed or truncated in the annotations provided by NCBI, here represented in darker color and labeled with the NCBI gene name. No annotation was found in NCBI for *B. innocent* and *B. hyodysenteriae*. All genes are represented 5’ to 3’; the scale measures nucleotides and is centered on the start codon of the “Sec.1” gene. (C) Structure alignments of the putative SECIS found downstream the TGA codon (underlined in red) in the two selenoproteins, “Sec.1” (left) and “Sec.2” (right). Alignments produced using Infernal [31] and visualized with RALEE [61]. See Fig 6 for RALEE colouring scheme.

We also detected three bacterial genomes with *SelD* and tRNA^Sec^, but without selenoprotein predictions from any known family. Although this may be caused by incomplete assemblies, it may suggest that these organisms use yet undiscovered selenoproteins. The three genomes (*Paenibacillus vortex V453* and the two strains *Brachyspira hampsonii 30446* and *30599*) were analyzed with a custom procedure to identify TGA-containing open reading frames (ORF) (Materials and Methods). The analysis revealed a putative novel selenoprotein in the *B. Hampsonii* genomes. The candidate selenoprotein is a small protein that has a thioredoxin domain (PF13192; “Thioredoxin 3”) with a short 5’ extension that contains a conserved Cys/Sec residue (Fig 7A). The Cys-containing homologues identified are annotated as “Redox-active disulfide protein 2”. We found this novel selenoprotein in all other *Brachyspira* genomes analyzed, which, in contrast to *B. Hampsonii*, we identified other selenoprotein families. All genomes had three genes from this protein family: a Cys-containing homologue and two selenoproteins. The three genes were always found forming a gene cluster (Fig 7B). The two putative selenoproteins had good candidate bacterial SECIS downstream their TGA codon (Fig 7C). One of the two selenoproteins (“Sec.1” in Fig 7A) lacked the redox-active motif (CXXC) in the thioredoxin domain (columns 61–64 in Fig 7A). Proteins from the “Redox-active disulfide protein 2” family are classified as oxidoreductases acting on a sulfur group of donors. A search in STRING database [65] revealed that the genes from this protein family commonly neighbour genes from other selenoprotein families such as thioredoxin reductases, alkyl hydrogen peroxide reductase, peroxiredoxins, and other oxidoreductases.

Results in eukaryotes are summarized in S8 Fig: in the genomes analyzed, tRNA^Sec^ correlated almost perfectly with the presence of Sec machinery factor EF-Sec and selenoprotein genes.

#### Novel Sec extinctions in arthropods

Most known metazoans encode selenoproteins with the exception of parasitic plant nematodes [12], and several insect orders, in which multiple Sec loss events have been described [6, 8, 9]. The analysis of the Secmarker predictions, however, provided a picture of much increased resolution of the distribution and evolution of the Sec trait in insects, and arthropods in general. Selenoproteins have been reported to be lost in *Lepidoptera* and *Hymenoptera* (i.e., no known species in these orders encode selenoproteins), and consistently, we did not find any other species from these orders encoding selenoproteins. *Coleoptera* were also assumed to entirely lack selenoproteins; however, we did find two coleopterans that encode selenoproteins. Selenoprotein losses have also been reported in some, but not all, *Diptera* and *Paraneoptera* species. Here we also found selenoproteinless species in *Trichoptera* and *Strepsiptera*. Finally, no arthropod outside insects have so far been reported to lack selenoproteins. Here, we report the genomes of two arachnids that lack selenoproteins. We next describe in additional detail these results (summarized in S9 Fig).

We did not find tRNA^Sec^, nor other Sec machinery factors, nor selenoproteins in the genome of the trichopteran *Limnephilus lunatus* (S9 Fig). Since *Trichoptera* is a sister group to *Lepidoptera* [66], our data suggest that selenoproteins could have been lost in the common ancestor of *Trichoptera* and *Lepidoptera*. Similarly, we did not find selenoproteins nor Sec machinery factors in the genome of *Mengenilla moldrzyki* (order *Strepsiptera*). Since all coleopterans analyzed to date lacked selenoproteins, it was assumed that a Sec loss event occurred at the root of the lineage [6, 8, 9]. However, we identified here two coleopterans with tRNA^Sec^, selenoproteins and a complete Sec machinery (S9 Fig). The genome of *Onthophagus taurus* contained two selenoprotein genes (*SPS2* and *SelK*), and *Nicrophorus vespilloides* contained a *SPS2* selenoprotein gene. All three genes have good candidate SECIS. From the phylogenetic topology of the available genomes from *Coleoptera*, based on [67], and from the phylogenetic location of the selenoprotein containing genomes, we infer that multiple independent Sec extinctions occurred in *Coleoptera*: in *Cucujiformia* (previously reported [6, 8, 9]), in the lineage leading to *Agrilus planipennis* (*Elateriformia*), and the lineage leading to *Priacma serrata* (*Archostemata*).

Outside insects, the genomes of the arachnids *Dermatophagoides farinae* and *Sarcoptes scabiei* also lacked selenoproteins and the Sec machinery factors (S9 Fig). These two species belong to *Acari*, a taxon of non-insect arthropods that include bulbs and mites, and they are the only two sequenced representatives from the order *Astigmata* (mites). Unlike selenoproteinless insects, these two genomes do not have a *SPS1* gene, the non-selenoprotein paralogue of *SPS2*. *SPS1* was predicted to emerge by gene duplication at the root of insects, as well as in other lineages independently [6]. In *Astigmata* it appears that SPS2 was lost without prior duplication to generate *SPS1*, analogously to the situation in selenoproteinless nematodes [12]. These are the two first non-insect arthropod genomes reported to have lost selenoproteins.

#### Intron-containing tRNA^Sec^

Among the genomes with more than one bona fide tRNA^Sec^ predictions is that of the crustacean *Daphnia pulex* (common water flea), in which we identified two copies. Strikingly, the two copies contain introns. Although introns are not rare in canonical tRNAs, only a single case has been reported for tRNA^Sec^. This was recently found in *Lokiarchaeota* [37], using Secmarker. Eukaryotic tRNA introns are generally short (14–60 nucleotides), and invariably interrupt the C-loop one base 3’ to the anticodon [68]. The introns in the two *D. pulex* tRNA^Sec^ genes are 25 and 16 nucleotides long, and are located in the expected position (S5 Fig). Both genes have a G in position 73. The sequences of the mature tRNAs differ only in two positions. Notably, these positions map to the T arm, and are predicted to form pairs in both genes. The presence of two mutations in the residues that form a pair suggest that a compensatory mutation occurred to maintain the integrity of the structure of the tRNA. However unusual, this strongly suggests that *D. pulex* possesses two functional copies of tRNA^Sec^, and that both have an intron.

#### Structure of the archaeal tRNA^Sec^

In spite of the low number of archaeal selenoprotein containing genomes analyzed, our results strongly support that tRNA^Sec^ in *archaea* has generally a 7 bp D-stem, one base pair longer than eukaryotes and bacteria, as reported by [36] after analyzing a smaller set of genomes. We observed the 7 bp D-stem in the 19 *Methanococcales* analyzed here. The only exception, with a canonical 6 bp D-stem, was *Methanopyrus kandleri* (S6 Fig) as already noted in [36]. The selenocysteine machinery in *Lokiarchaeota*, the most recently identified Sec-containing lineage in archaea, includes a tRNA^Sec^ with a 7 bp D-stem and an intron in the T arm [37].

#### Conservation of the eukaryotic tRNA^Sec^

**Fig 8:**
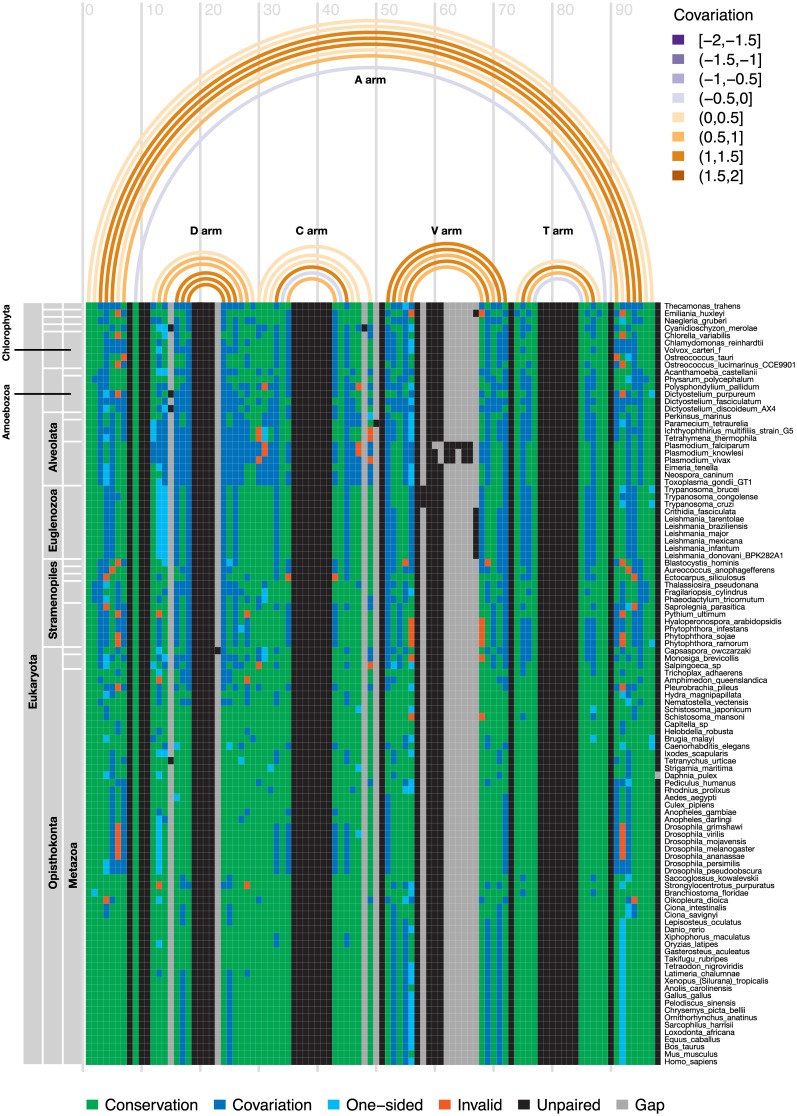
Structure conservation of tRNA^Sec^ across eukaryotes. Arc diagram of eukaryotic tRNA^Sec^ displaying covariation information. The arcs link the residues that form each pair in the tRNA secondary structure, and are colored according to the covariation (top legend). The blocks correspond to the structural alignment of the tRNA^Sec^ sequences, and are colored according to the covariation in each sequence (bottom legend). The labels on the right indicate the name of the species, which are clustered by their phylogeny (left panel). Plot produced with R-chie [69]. In R-chie the covariation values (top legend) have a range of [-2, 2], where -2 is a complete lack of pairing potential and sequence conservation, 0 is complete sequence conservation regardless of pairing potential, and 2 is a complete lack of sequence conservation but maintaining pairing.

We evaluated the conservation of the tRNA^Sec^ structure across eukaryotes. We used the program R-chie [69] to analyze the structural alignment containing the top scoring predictions in the benchmark set. The alignment largely supports the eukaryotic tRNA^Sec^ structural model [32, 33], showing covariation of nucleotide pairs (i.e., variation of the two nucleotides that form a pair keeping the canonical base pairing) in all tRNA arms. The V arm showed the highest level of variability, and the anticodon arm, the lowest (Fig 8). Based on a larger alignment including the 553 eukaryotic top scoring G73 tRNA^Sec^ candidates, there were only six positions, besides the anticodon triplet and the residue 73, 100% conserved across all species: G18 and G19 in the D-loop, U33 in the anticodon loop, U55 in the T-loop, C61 in the T-stem and C66 in the acceptor stem. Overall conservation, measured as the average of the conservation at each position, was higher in unpaired residues in loops and the linker region between acceptor and D arms (92%) than in paired residues in the stems (82%).

#### tRNA^Sec^ with anticodon CUA

A remarkable finding was recently reported in [56], where the authors described bacterial organisms that code for Sec with codons other than UGA. In these species, tRNA^Sec^ has an anticodon different than UCA, and accordingly, there are selenoprotein genes carrying a matching codon at the Sec site. We identified three such tRNAs in our set of prokaryotic genomes. The genomes belonged to the *Geodermatophilaceae* family, and, as reported in [56], their tRNA^Sec^ had the anticodon CUA. Secmarker correctly identified these tRNA^Sec^ variants. We used Selenoprofiles [24] to predict selenoprotein genes in those three genomes, and in addition to the formate dehydrogenases (FDHs) and UGSC-motif selenoproteins reported in [56], we identified a gene encoding an alkyl hydroperoxide reductase (AhpC) selenoprotein with a Sec-TAG codon in the genome of *Blastococcus saxobsidens DD2* (S7 Fig).

## Discussion

Prediction of tRNA^Sec^ has never received wide attention, possibly because of the low number of selenoprotein genes. Thus, while general purpose tRNA detection methods, such as tRNAscan-SE and aragorn have been thoroughly benchmarked for canonical tRNAs, this is not the case for tRNA^Sec^ predictions–the tRNAscan-SE authors explicitly citing as a reason the low number of tRNA^Sec^ sequences available [26]. Indeed, among the more than 12,000 tRNA genes in tRNAdb [42], only 46 correspond to tRNA^Sec^.

Here, we built on the unique structural features of tRNA^Sec^ to create covariance models that allow Secmarker to identify tRNA^Sec^ genes with great accuracy. In addition to the intrinsic biological interest of refining the tRNA^Sec^ structural features and improving tRNA^Sec^ predictions, thus contributing to better genome annotations, accurate prediction of tRNA^Sec^ genes has the additional benefit of serving as marker of Sec utilization and selenoprotein encoding capacity in genomes. Since annotation of selenoprotein genes requires dedicated effort, pre-scanning the genome with Secmarker, which is reasonably fast (∼4 Mb/s), helps to allocate this effort only when needed.

Because, unlike the rest of amino acids, which are present in virtually all living species, Sec is only present in species encoding selenoproteins (to date about one quarter of all species with sequenced genomes), we were able to design a reliable benchmark for tRNA^Sec^ predictions. Indeed, tRNA^Sec^ predictions in selenoproteinless genomes are necessarily false positives, while lack of predictions in selenoprotein containing genomes denote false negatives. No equivalent benchmark can be implemented to evaluate predictions of tRNAs for other amino acids. As a marker of Sec utilization, Secmarker performs flawlessly; in our benchmark set, it predicted tRNA^Sec^ genes in all genomes encoding selenoproteins, and it did not produce predictions in any of the genomes lacking them. In contrast, tRNAscan-SE and aragorn failed to produce predictions in genomes known to encode selenoproteins, while producing predictions in genomes known to lack them.

This accuracy at the “genome level” is only an approximation, however, to the real accuracy of tRNA^Sec^ prediction programs. Indeed, a tRNA^Sec^ prediction in a selenoprotein containing genomes, while accurate as a marker of Sec utilization, could actually be a false positive if the wrong locus (or loci) are predicted, leading also to a false negative if, in addition, the correct tRNA^Sec^ is not predicted. This is often the case for aragorn and tRNAscan-SE. For instance, Secmarker failed to predict tRNA^Sec^ in the selenoprotein containing genome of *P. capsici* because the tRNA^Sec^ gene is missing from the current assembly, as revealed by the analysis of the raw reads available for this genome. However, aragorn predicted tRNA^Sec^ candidates, and, as markers of Sec utilization, they would be considered correct in our benchmark. However, manual inspection of the candidates revealed that these predictions do not possess the features of *bona fide* tRNA^Sec^. In fact, the secondary structure of the two candidates predicted by aragorn in *P. capsici* did not fit the tRNA^Sec^ model (S1 Text).

Evaluating the accuracy of the programs at the gene level is, however, challenging, since for most genomes we do not know the functional tRNA^Sec^ genes. Nevertheless, our results strongly suggest that Secmarker has a much lower false positive rate than tRNAscan-SE and aragorn. First, the average tRNAscan-SE genes predicted per genome is 1.7 for Secmarker, 20 for aragorn and 47 for tRNAscan-SE. Since, with a few exceptions, genomes encode at the most one single tRNA^Sec^ gene, the majority of tRNA^Sec^ aragorn and tRNAscan-SE predictions are actually false positives. Secmarker can also produce false positive predictions. We can attempt to estimate their ratio from the analysis of the Secmarker results in the full set of genomes. Ignoring non G73 predictions, that can be trivially filtered out, Secmarker predicted 154 tRNA^Sec^ candidates in 80 genomes (the 145 mentioned in Results plus 9 identical copies reported by Secmarker in those 80 genomes), with mutations destabilizing the tRNA^Sec^ structure when compared to the top scoring prediction in the same genome. Thus, we estimated the lower boundary for the Secmarker false positive ratio to be less than 5% (154 out 3213 total G73 predictions). We do not believe this lower boundary to depart too much from the actual false positive ratio, since Secmarker most often predicts a single tRNA^Sec^ gene in selenoprotein containing genomes. We believe the false negative ratio (i.e., the failure of Secmarker to predict the actual tRNA^Sec^ gene) to be negligible, since analysis of the selenoprotein containing genomes from the benchmarking set in which Secmarker failed to predict a tRNA^Sec^ gene revealed in all cases that the gene was missing from the analyzed genome assembly.

The accurate predictions of tRNA^Sec^ by Secmarker allowed us to reclassify a number of genomes thought to lack selenoproteins, as selenoprotein containing instead, as well as to re-evaluate the phylogenetic distribution of selenoprotein encoding genomes within insects. Thus, we identified two novel selenoproteinless insect orders, *Trichoptera* and *Strepsiptera*. Conversely, we found selenoproteins in two coleopterans, which were previously assumed to lack selenoproteins. We also found two selenoproteinless arachnid species, revealing the first selenoprotein extinction observed in non-insect arthropods. Secmarker predictions also led to the identification of a novel bacterial selenoprotein family. Finally, they allowed us to consolidate recent findings, as well as to produce novel insights, about tRNA^Sec^. Thus, our results support the tRNA^Sec^ archaeal fold, initially proposed based on a few sequences [36], and help to refine the novel bacterial fold recently reported [56]. In addition, we have traced the evolutionary history of the duplication and pseudogenization of tRNA^Sec^ occurred at the root of hominids, and report two intron containing tRNA^Sec^ genes occurring in *Daphnia*–the first eukaryotic intron-containing tRNA^Sec^ reported. Finally, in contrast to previous reports, we have identified a number of genomes that contain multiple tRNA^Sec^ copies likely to be functional, since they exhibit compensating mutations. Notably, we identified three eukaryotic genomes with four non-identical tRNA^Sec^ copies with compensating mutations. Since these genomes are phylogenetically diverse (the common house spider, a diatom and a lancelet), the duplicated tRNA^Sec^ are likely to have independent origins. Their biological significance is unclear, since the genomes of these organisms do not encode particularly large numbers of selenoproteins compared to the genomes of organisms from the same taxa.

tRNAs with a non-canonical structure can be responsible for alterations in the universal genetic code (e.g., selenocysteine [70] and pyrrolysine [71]), but they are likely to be missed or misannotated. Recent studies have identified novel uncommon tRNA structures [72, 73], revealing additional complexity in the genetic code. The use of dedicated tools, as we shown here, can be useful for the proper identification and annotation of non-canonical tRNAs.

In summary, we described here the development and validation of Secmarker, a tool to predict tRNA^Sec^. The analysis of its predictions across thousands of genomes revealed a number of insights, ultimately contributing to our understanding of tRNA^Sec^ and selenoproteins–one of the most fascinating class of proteins.

## Materials and methods

Secmarker is a novel tRNA^Sec^ detection pipeline based on covariance models (CM). It includes three manually curated CMs for tRNA^Sec^. Each model corresponds to a domain of life (archaea, bacteria and eukaryotes) and incorporates its characteristic structural features. The program scans a nucleotide sequence with the three models using cmsearch from the Infernal package (v1.1.1) [31]. After processing and filtering the hits by Infernal, the program produces a graphical output showing the tRNA^Sec^ secondary structure (Fig 1).

### Secmarker availability

**Fig 9:**
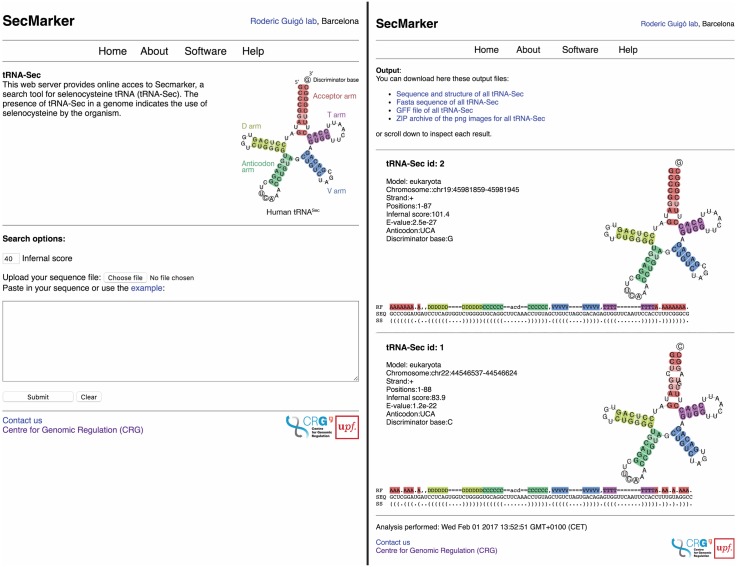
Secmarker web server. Two snapshots showing the input form (left) and the output page (right). The results shown correspond to the two human tRNA^Sec^.

Secmarker is available for online analysis at http://secmarker.crg.cat (Fig 9). The web server accepts sequences up to 100Mb, and runs at a search speed of ∼4 Mb/s. After processing and filtering the candidates produced by Infernal, the program identifies their discriminator base and produces a graphical output showing the tRNA^Sec^ cloverleaf secondary structure. The program can also be downloaded, installed, and run locally. Secmarker is written in python and requires a local installation of the Infernal package [31] (version 1.1.1, available at http://eddylab.org/infernal/) and the ViennaRNA package [50] (tested on version 2.1, available at http://www.tbi.univie.ac.at/RNA). Secmarker has been tested on python 2.6.6 and 2.7.10.

### tRNA^Sec^ covariance models

CMs are ‘a specialized type of stochastic context-free grammar’ [31]. Infernal [31] can be used to build a CM from a multiple nucleotide sequence alignment with structural annotation. The sequences used to build the three models were obtained from the Rfam database [74] (RF01852, tRNA^Sec^). Here, it is important to mention that Rfam provides a single model for tRNA^Sec^. However, given the structural differences of tRNA^Sec^ between the three domains of life, we built three independent, domain-specific models. In order to build the models, first, the Rfam tRNA^Sec^ sequences were downloaded and clustered according to their taxonomic domain, using the species identifier. Then, tRNAscan-SE [26] was used to filter out sequences that did not match the eukaryotic or prokaryotic models, according to tRNAscan-SE labels “SeC(e)” and “SeC(p)”, respectively. With the remaining sequences, a recursive procedure using RNAfold from the Vienna package [50], and cmalign and cmbuild from the Infernal package, was designed to iteratively align the sequences based on their predicted structure. Finally, sequences with an anticodon different than UCA were discarded. The alignments used to build the models with cmbuild contained 10, 140 and 251 sequences for archaea, eukaryota and bacteria, respectively. The alignments and covariance models used by Secmarker are provided in S1 File.

### Search phase and filtering

The target nucleotide sequence is scanned with the three CMs using cmsearch [31], as first step. The default bit score cut-off for cmsearch is 40, but this can be set by the user using the -T option. This threshold was set upon confirmation that cmsearch did not miss any true positive in the benchmark set. Often, the same locus is identified by more than one model. Overlapping hits are thus removed, keeping for each locus only the hit with the highest bit score. The resulting hits are processed to identify the anticodon triplet, the boundaries of each tRNA arm and the position 73 (see next section). By default any anticodon is accepted, although hits with a anticodon different than UCA are filtered through a more stringent bit score threshold (55). The final candidates are filtered through a custom procedure designed to identify the most common false positives: hits with shorter or missing arms. The tRNA^Sec^ candidates in the output are labeled according to the model (eukaryotic, archaeal or bacterial) with the highest bit score by cmsearch.

### Discriminator base identification

Secmarker runs a procedure to identify the position 73, the discriminator base, in tRNA^Sec^, exploiting the length of 13 nt of the AT-stem in this family of tRNAs. This position is not included in our models, so it is not considered in the search phase. In order to identify the position 73, the program first identifies the position 61 (numbering based on [35]), and then retrieves the 14th base 3’ from that position, if that nucleotide is present in the input sequence. Since the total length of the sequence predicted by Infernal at the search phase, could vary according to the number of pairs in the acceptor arm, this procedure is independent of the number of pairs in the acceptor stem.

### Graphical tRNA^Sec^ structure

Secmarker produces a graphical output representing the secondary structure of the predicted tRNA^Sec^ genes (Fig 1). The tRNA structure is represented in its cloverleaf form, with the different nucleotide pairs colored according to the arm. Wobble pairs (GU and UG) are indicated with a faint color. The nucleotides in the anticodon triplet (normally UCA) are circled. The discriminator base, is also circled, if detected. The graphical output can be activated using the flag -plot, which by default is off. In order to produce the graphical output, Secmarker requires a local installation of the program RNAplot from the Vienna package (tested on version 2.1.1) [50].

### Benchmarking

In order to test the prediction of tRNA^Sec^, we used a set of 641 sequenced genomes (212 eukaryotes, 217 bacteria and 212 archaea). We had previously analyzed the bacterial and eukaryotic organisms in this set for the presence of the Sec utilization trait and selenoproteins [6]. The set of archaeal genomes not analyzed in [6], was obtained from NCBI. Sec utilization was predicted in these species based on the presence of the genes for *selD* and *EF-sec*, which were annotated using Selenoprofiles [24].

Secmarker, aragorn v1.2 [27], tRNAscan-SE v1.23 [26] and Infernal 1.1 [31] with RF01852 (Rfam tRNA-Sec) were used to predict tRNA^Sec^ in the genomic sequences. Aragorn was executed using the -t flag (predict tRNA only). For prokaryotic sequences, tRNAscan-SE was executed with -B flag. The single tRNA-Sec model RF01852 was used using the parameters recommended in the curation page (http://rfam.xfam.org/family/RF01852#tabview=tab9), ‘cmsearch –nohmmonly -T 25.39’. The results were then parsed to exclude those hits with score lower than 47.0 (“gathering threshold”). Bacterial tRNA^Sec^ genes predicted in eukaryotic genomes were assumed to originate from bacterial contamination in the eukaryotic genome assemblies. We could filter out such cases from the output of tRNAscan-SE and Secmarker. All programs were executed in a SGE distributed cluster using a single cpu with 12Gb of memory available.

### Identification of TGA-containing ORFs

We implemented a procedure to identify TGA-containing ORFs in prokaryotic genomes. The procedure was based on the modification of an existing annotation of protein coding genes. The genes included in the annotation were extended at both ends, using the same frame of translation, up to a stop codon different than TGA. All in-frame TGA codons were included in the extensions. The amino acid sequence of the TGA-containing ORFs were analyzed for sequence conservation using Blastp [75] against the protein database UniRef90 from UniProtKB. Since all selenoprotein families have Cys-containing homologues (non-selenoprotein genes with a Cys residue at the homologous Sec position), we expected any selenoprotein gene to show TGA/Cys pairs in the Blast alignments. We parsed the Blast outputs and selected those ORFs that produced three or more hits with a TGA aligned to a Cys residue. The selected ORFs were analyzed further. For each ORF, a profile alignment, containing the TGA-containing sequence and the Cys homologues identified by Blast, was build and used to scan a set of bacterial genomes with Selenoprofiles [24].

### Public sequencing datasets

Pol III chip-seq data analyzed in this work was produced in [59]. We downloaded the fastq files corresponding to human liver samples (ERR039133 and ERR039141) from ArrayExpress (https://www.ebi.ac.uk/arrayexpress/E-MTAB-958; accession: E-MTAB-958;). The fastq files were processed using our in-house chip-seq pipeline (https://github.com/guigolab/chip-nf). *Phytophthora capsici* Genome Sequencing Illumina HiSeq 2000 reads were downloaded from NCBI SRA (http://www.ncbi.nlm.nih.gov/sra), accessions: SRR943799 and SRR945695, and analyzed with Secmarker. The following reads contained the full sequence of a eukaryotic tRNA^Sec^ gene: SRR943799.568178, SRR943799.262468, SRR943799.84635, SRR945695.19108665, SRR945695.14526540, SRR945695.2975118.

## Supporting information

S1 Fig Structural alignment of bacterial tRNA^Sec^ candidates with a 7 base pairs acceptor stem. The alignment contains 52 tRNA^Sec^ sequences identified in this study, including the 47 top scoring candidates plus five gene copies (indicated with a star), with an unusually short 7 bp acceptor stem. The acceptor stem is delimited by the T-stem (brown) and the residue G73 (the 4th residue from the right), and has 7 pairs (grey) in all sequences. Positions where bulged nucleotides can be observed are numbered in red on top of the alignment. The nucleotides numbering is based in [35]. The sequences were aligned using Infernal [31] and visualized with RALEE [61]. RALEE highlights the nucleotides that are paired according to the consensus secondary structure (second line from the bottom, SS_cons) of the alignment, and that also respect the standard pairing rules.
(TIF)

S2 Fig Cloverleaf structure of bacterial tRNA^Sec^ candidates with a 7 base pairs acceptor stem. Inferred secondary structure of bacterial tRNA^Sec^ candidates. The structures have a 7 bp acceptor stem (one pair shorter than the canonical bacterial tRNA^Sec^) and show a bulged nucleotide in different positions in the acceptor stem. They are classified in four types (columns A-D) according to the bulged nucleotide in the acceptor arm: (A) position 3a, (B) 4a, and (C) 5a; (D) has an extra nucleotide in position 7a, in the linker region between the acceptor stem and D-stem. Other bulged nucleotides are also indicated with red numbers. Numbering based on [35]. Genes *selA*, *selB* and *selD* were often found in proximity to tRNA^Sec^, and are shown above the corresponding structure.
(TIF)

S3 Fig Multiple tRNA^Sec^ predictions in genomes. Distribution of scores obtained in non-identical tRNA^Sec^ predictions (3,226) for the top scoring candidates (“top”) and for the multiple copies (“copies”). The predictions were split according to the residue in position 73 into the following categories: G73, non-G73 and G73CM (copies with G73 and with compensatory mutations when compared to the top scoring one).
(TIF)

S4 Fig AhpC protein in *Blastococcus saxobsidens DD2* incorporates Sec in response to a UAG codon. (A) Multiple sequence alignment of bacterial AhpC proteins. The selenocysteine residue (red) in *B. Saxobsidens DD2* (top) corresponds to a UAG codon in the genome sequence. (B) The AhpC UAG-Sec codon (underlined in red) followed by a bSECIS secondary structure, predicted with RNAfold [50]. (C)The tRNA^Sec^ in *B. Saxobsidens* has a CUA anticodon, complementary to the UAG codon. Protein identifiers: *Sphaerobacter thermophilus* D1CAV3_SPHTD, *Xanthobacter autotrophicus* A7IJH6_XANP2, *Ktedonobacter racemifer* D6TT72_9CHLR, *Rhodopirellula sallentina* M5U546_9PLAN, *Hirschia baltica* C6XML7_HIRBI.
(TIF)

S5 Fig C-loop intron-containing tRNA^Sec^ genes in *Daphnia pulex*. Structural alignment of the two intron-containing tRNA^Sec^ genes identified in this study, and the cloverleaf structure (including the longest intron). The boundaries of the introns are indicated by the dashed lines. The rightmost position of the alignment corresponds to the discriminator base. The sequences were aligned using Infernal [31] and visualized with RALEE [61]. See S1 Fig caption for RALEE coloring scheme.
(TIF)

S6 Fig Structural alignment of archaeal tRNA^Sec^. The 20 archaeal tRNA^Sec^ sequences identified in this study are included. Note the 7 bp D-stem (light blue) in all sequences, with the exception *M. kandleri*. The sequences were aligned using Infernal [31], and visualized with RALEE [61]. See S1 Fig caption for RALEE coloring scheme.
(TIF)

S7 Fig Multiple sequence alignment of tRNA^Sec^ candidates in *Fragilariopsis cylindrus*. The eleven tRNA^Sec^ candidate sequences in the *F. cylindrus* genome, including the 100 nt in the flanking regions, are shown. The tRNA boundaries correspond to the positions 101–187. The secondary structure is represented below the tRNA region. Five of the sequences (6, 7, 10, 11 and 9) exhibit compensatory mutations (green) compared to the top scoring candidate (1, top), although two of them (11 and 9) have a mutation that produces a mismatch in one of the pairs (red). The remaining mutations (white) would not affect the pairing potential of the sequence.
(TIF)

S8 Fig Correlation of tRNA^Sec^ and selenoproteins across eukaryotes. Sunburst diagram showing the eukaryotic genomes in our set. The presence of tRNA^Sec^ (black dot) and EF-Sec (white dot) genes is indicated in the terminal nodes, and the number of selenoproteins is indicated by a black bar. The length of the bar is proportional to the number of selenoproteins. Some nodes were collapsed based on the presence of tRNA^Sec^. Those nodes include a number in parentheses, indicating the number of species collapsed. In the collapsed nodes, the average number of selenoproteins was computed, and a white dot indicates that all species contain EF-Sec genes. The phylogeny was obtained from NCBI taxonomy.
(TIF)

S9 Fig Sec extinctions in arthropods. Species tree including a subset of the arthropod genomes analyzed in this work. The shaded boxes indicate known (dark grey) and novel (red) Sec extinctions. Each species is annotated with the presence of tRNA^Sec^, the protein factors of the Sec machinery (including the selenoprotein SPS2-Sec), and SPS1 genes. SPS1-Arg corresponds to SPS genes with an arginine codon at the homologous Sec position, and SPS1-rt corresponds to SPS genes with a UGA codon, in which a readthrough event occurs but the inserted amino acid is not known (see [6]). The black horizontal bar indicates the number of selenoproteins. The topology of the *Coleoptera* lineage was adapted according to [67].
(TIF)

S1 File Infernal models. The file S1_File.tar contains the three alignments (eukaryota.stk, bacteria.stk and archaea.stk), the tRNAsec_db.stk file (the concatenation of the three alignments), and the tRNAsec_db.cm file (the infernal cmbuild model). tRNAsec_db.cm was generated with infernal 1.1.1 using the following parameters: “cmbuild --hand tRNAsec_db.cm tRNAsec_db.stk” and “cmcalibrate tRNAsec_db.cm”.
(TAR)

S1 Text Supplementary information. (PDF)

S1 Table Full scientific names of prokaryotic genomes used in the benchmark set. (CSV)
