# Supplementary figures and images for: Computational identification of the selenocysteine tRNA (tRNASec) in genomes
Source: PLoS Comput Biol. 2017 Feb 13;13(2):e1005383. doi: 10.1371/journal.pcbi.1005383 (PMC5330540; doi:10.1371/journal.pcbi.1005383)

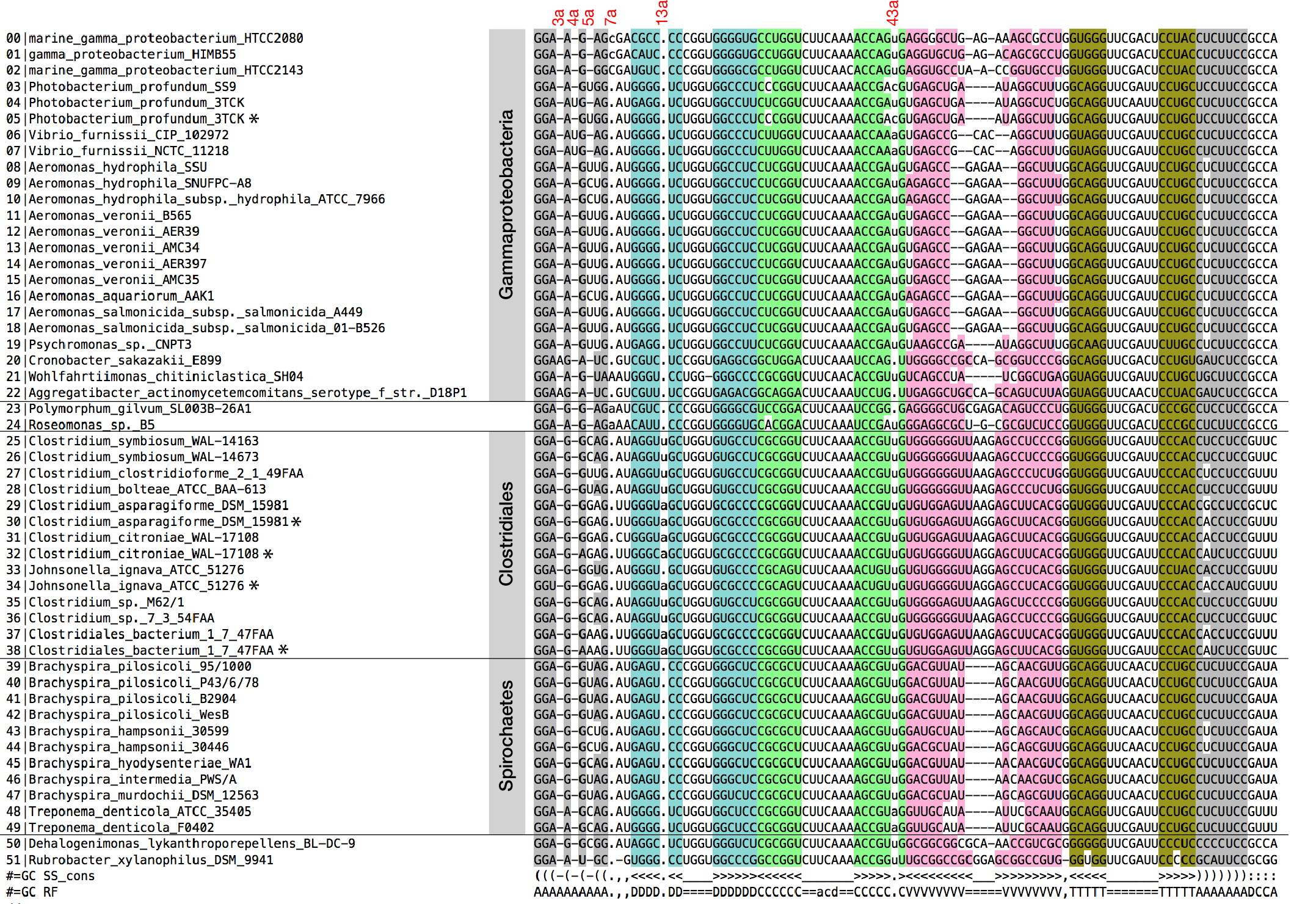

Supplement: S1 Fig — The alignment contains 52 tRNASec sequences identified in this study, including the 47 top scoring candidates plus five gene copies (indicated with a star), with an unusually short 7 bp acceptor stem. The acceptor stem is delimited by the T-stem (brown) and the residue G73 (the 4th residue from the right), and has 7 pairs (grey) in all sequences. Positions where bulged nucleotides can be observed are numbered in red on top of the alignment. The nucleotides numbering is based in [35]. The sequences were aligned using Infernal [31] and visualized with RALEE [61]. RALEE highlights the nucleotides that are paired according to the consensus secondary structure (second line from the bottom, SS_cons) of the alignment, and that also respect the standard pairing rules. (TIF) [file pcbi.1005383.s001.tif]

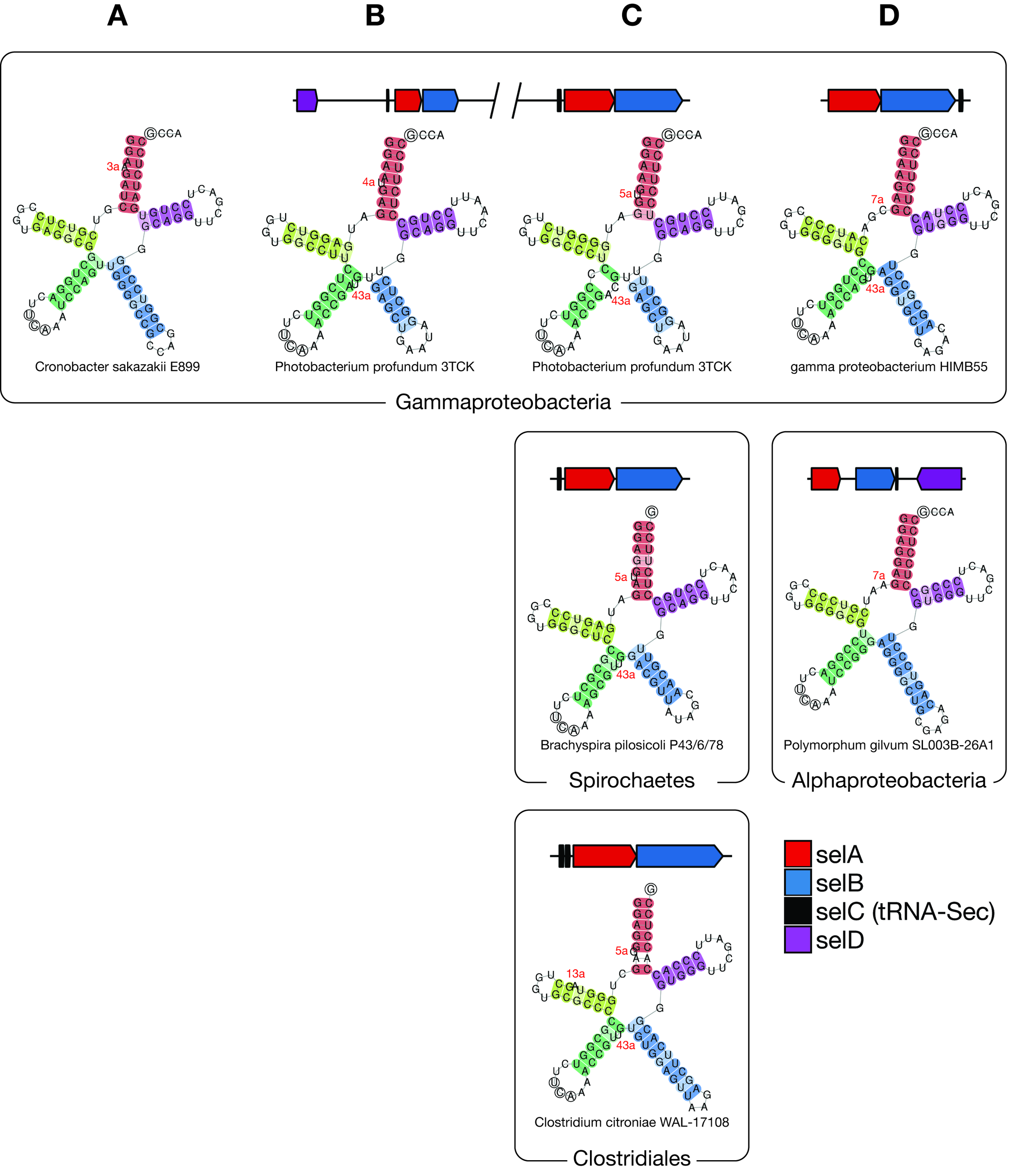

Supplement: S2 Fig — Inferred secondary structure of bacterial tRNASec candidates. The structures have a 7 bp acceptor stem (one pair shorter than the canonical bacterial tRNASec) and show a bulged nucleotide in different positions in the acceptor stem. They are classified in four types (columns A-D) according to the bulged nucleotide in the acceptor arm: (A) position 3a, (B) 4a, and (C) 5a; (D) has an extra nucleotide in position 7a, in the linker region between the acceptor stem and D-stem. Other bulged nucleotides are also indicated with red numbers. Numbering based on [35]. Genes selA, selB and selD were often found in proximity to tRNASec, and are shown above the corresponding structure. (TIF) [file pcbi.1005383.s002.tif]

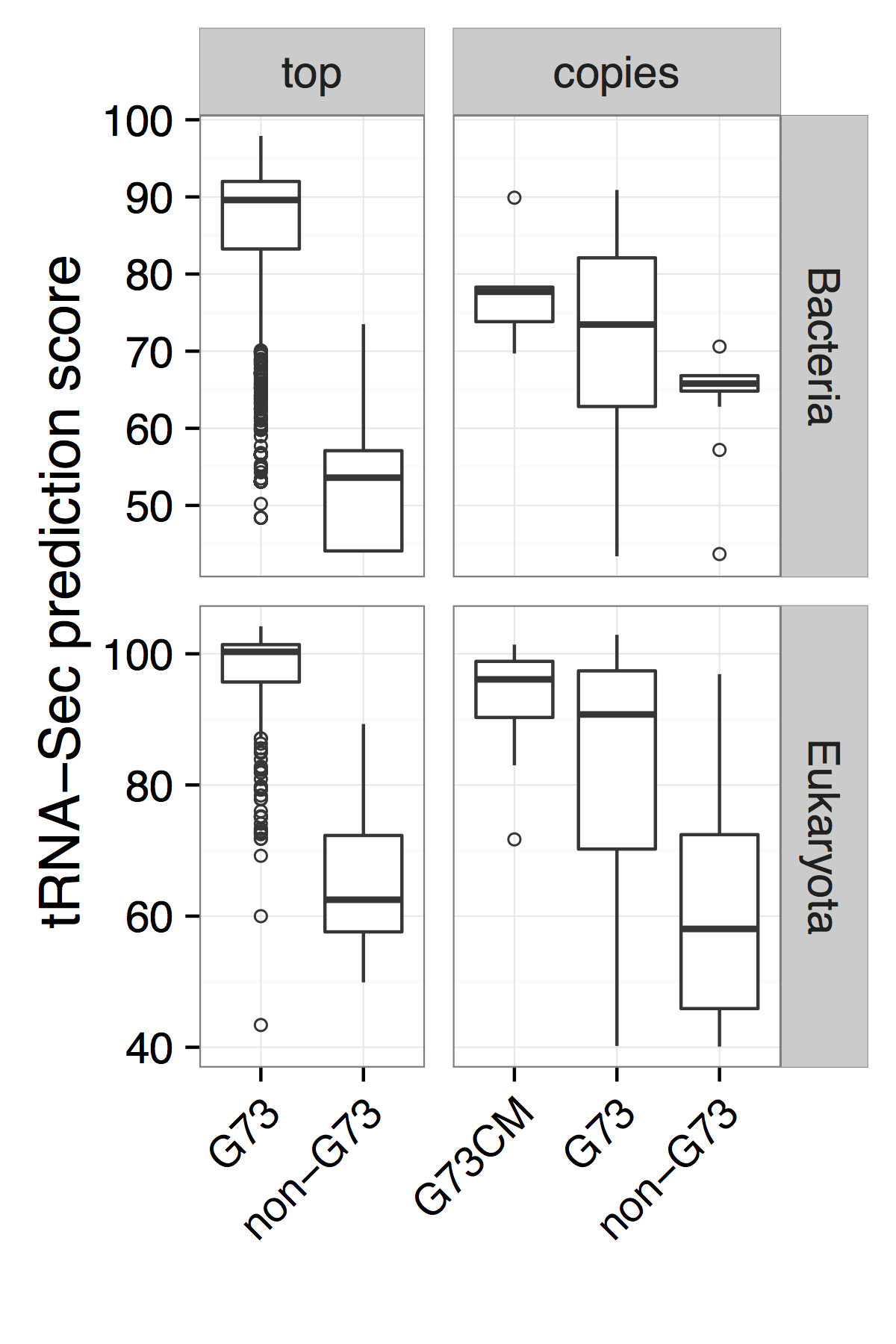

Supplement: S3 Fig — Distribution of scores obtained in non-identical tRNASec predictions (3,226) for the top scoring candidates (“top”) and for the multiple copies (“copies”). The predictions were split according to the residue in position 73 into the following categories: G73, non-G73 and G73CM (copies with G73 and with compensatory mutations when compared to the top scoring one). (TIF) [file pcbi.1005383.s003.tif]

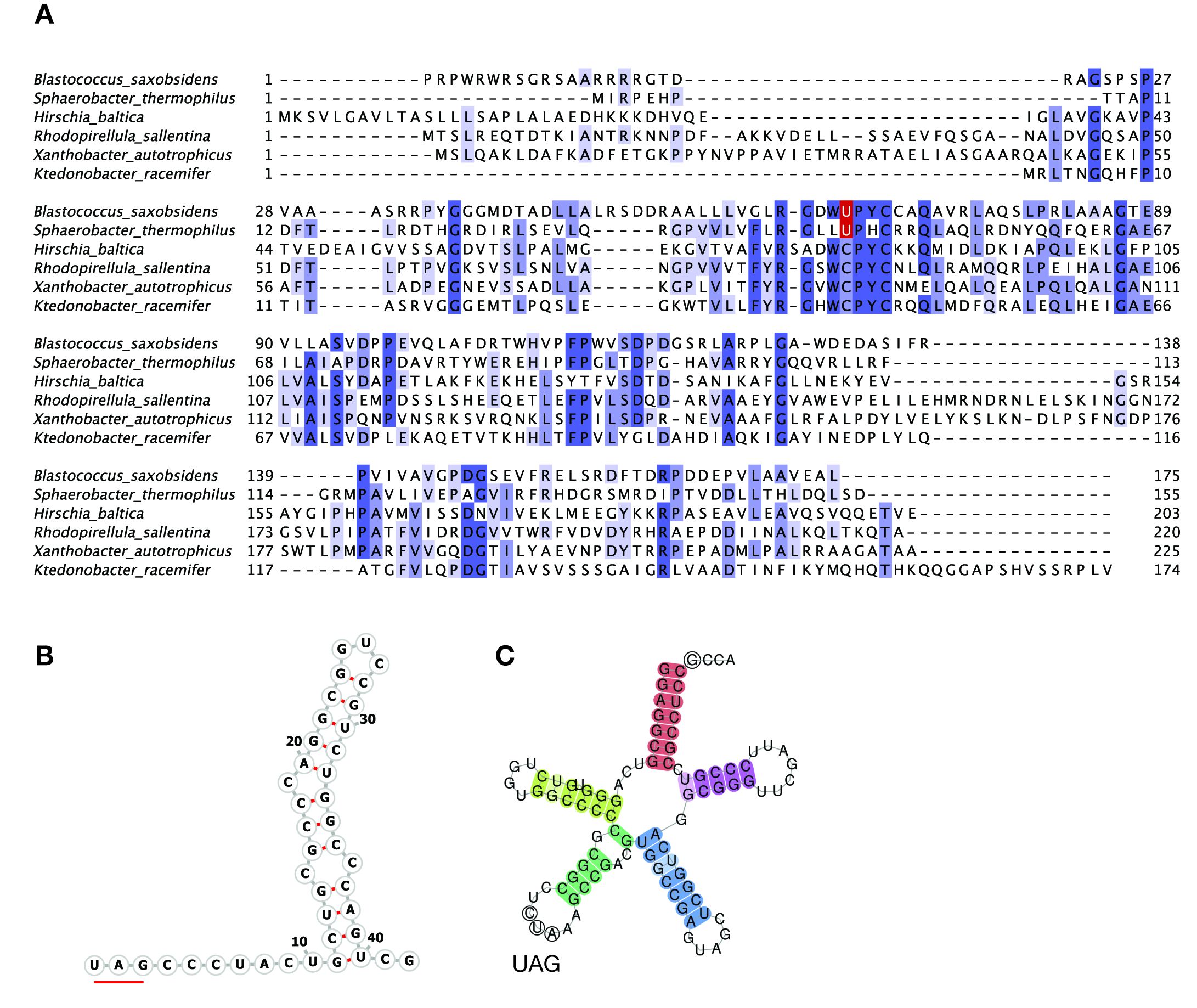

Supplement: S4 Fig — (A) Multiple sequence alignment of bacterial AhpC proteins. The selenocysteine residue (red) in B. Saxobsidens DD2 (top) corresponds to a UAG codon in the genome sequence. (B) The AhpC UAG-Sec codon (underlined in red) followed by a bSECIS secondary structure, predicted with RNAfold [50]. (C)The tRNASec in B. Saxobsidens has a CUA anticodon, complementary to the UAG codon. Protein identifiers: Sphaerobacter thermophilus D1CAV3_SPHTD, Xanthobacter autotrophicus A7IJH6_XANP2, Ktedonobacter racemifer D6TT72_9CHLR, Rhodopirellula sallentina M5U546_9PLAN, Hirschia baltica C6XML7_HIRBI. (TIF) [file pcbi.1005383.s004.tif]

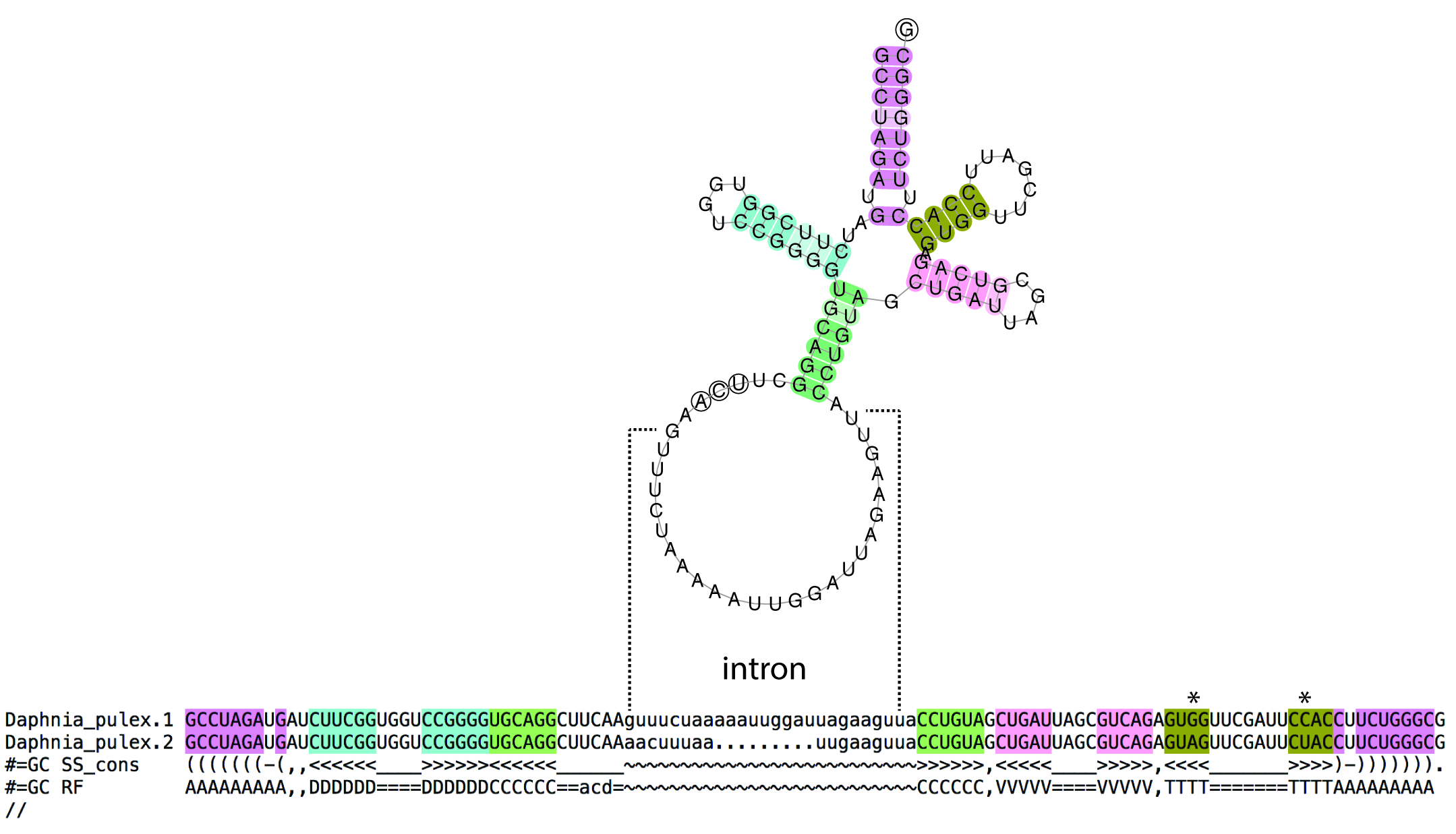

Supplement: S5 Fig — Structural alignment of the two intron-containing tRNASec genes identified in this study, and the cloverleaf structure (including the longest intron). The boundaries of the introns are indicated by the dashed lines. The rightmost position of the alignment corresponds to the discriminator base. The sequences were aligned using Infernal [31] and visualized with RALEE [61]. See S1 Fig caption for RALEE coloring scheme. (TIF) [file pcbi.1005383.s005.tif]

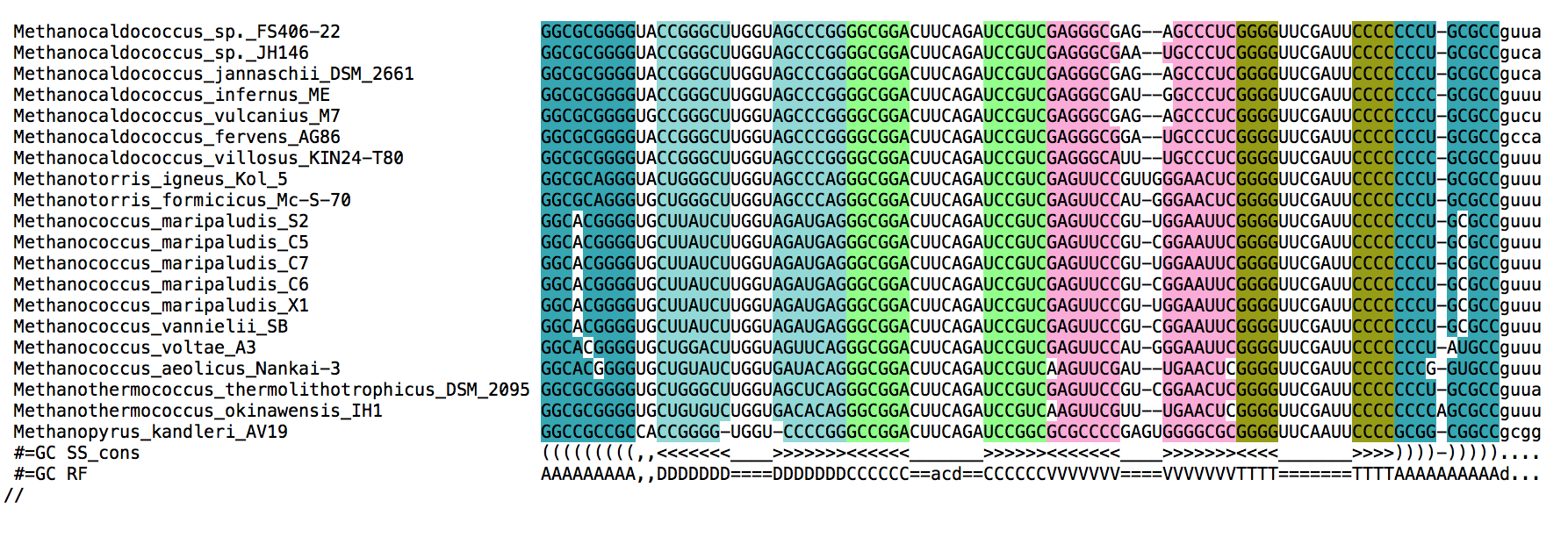

Supplement: S6 Fig — The 20 archaeal tRNASec sequences identified in this study are included. Note the 7 bp D-stem (light blue) in all sequences, with the exception M. kandleri. The sequences were aligned using Infernal [31], and visualized with RALEE [61]. See S1 Fig caption for RALEE coloring scheme. (TIF) [file pcbi.1005383.s006.tif]

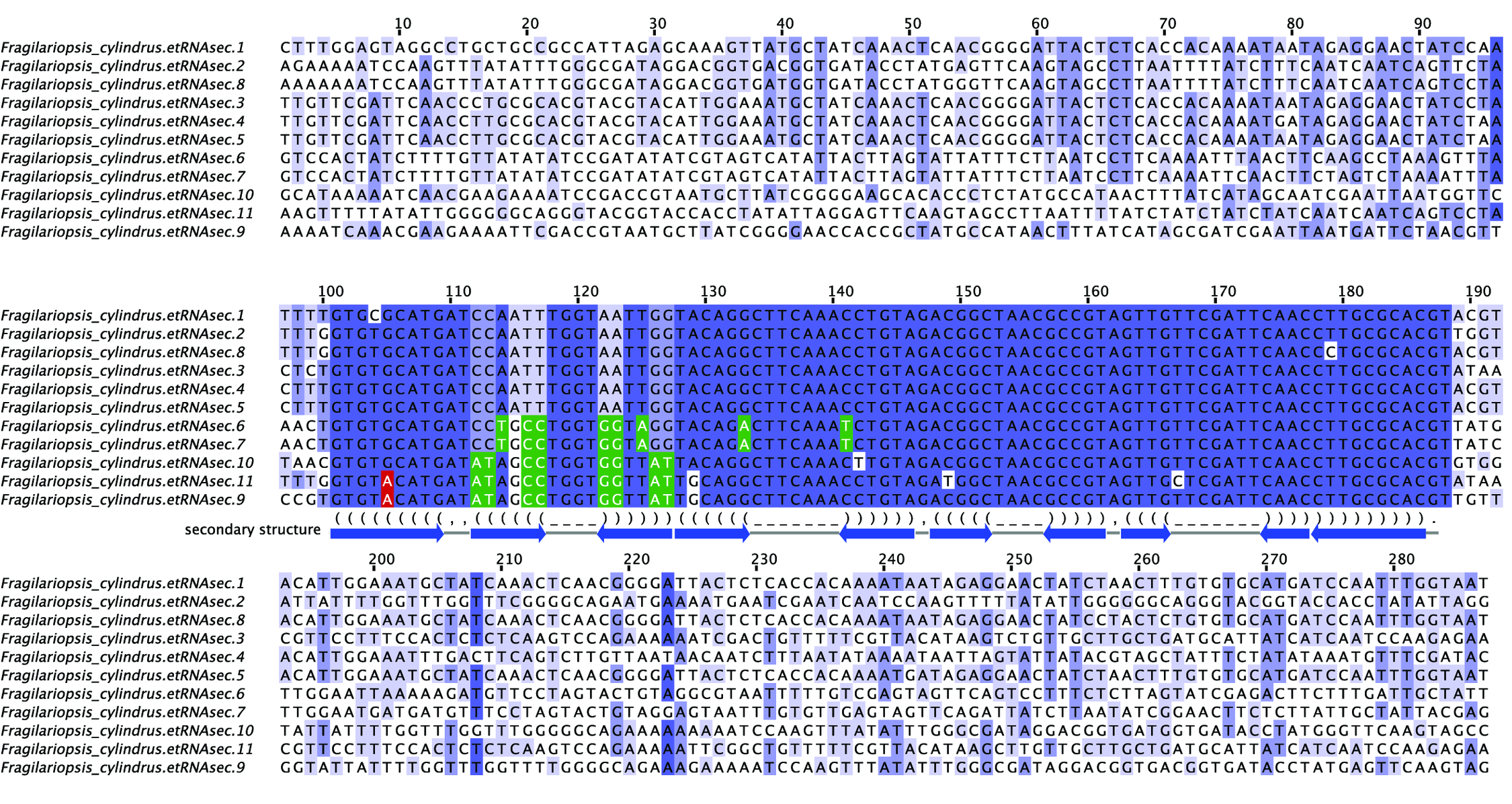

Supplement: S7 Fig — The eleven tRNASec candidate sequences in the F. cylindrus genome, including the 100 nt in the flanking regions, are shown. The tRNA boundaries correspond to the positions 101–187. The secondary structure is represented below the tRNA region. Five of the sequences (6, 7, 10, 11 and 9) exhibit compensatory mutations (green) compared to the top scoring candidate (1, top), although two of them (11 and 9) have a mutation that produces a mismatch in one of the pairs (red). The remaining mutations (white) would not affect the pairing potential of the sequence. (TIF) [file pcbi.1005383.s007.tif]

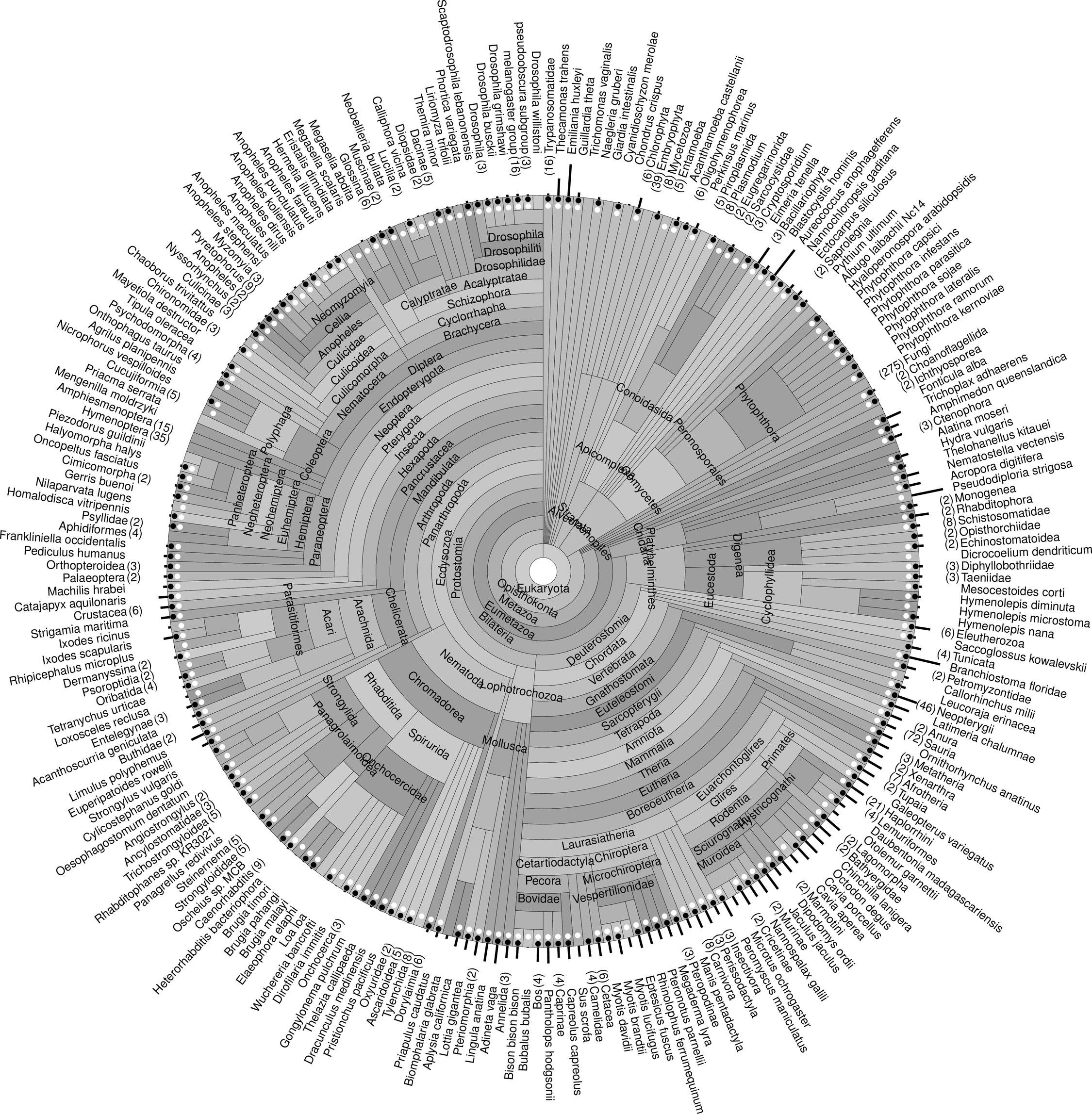

Supplement: S8 Fig — Sunburst diagram showing the eukaryotic genomes in our set. The presence of tRNASec (black dot) and EF-Sec (white dot) genes is indicated in the terminal nodes, and the number of selenoproteins is indicated by a black bar. The length of the bar is proportional to the number of selenoproteins. Some nodes were collapsed based on the presence of tRNASec. Those nodes include a number in parentheses, indicating the number of species collapsed. In the collapsed nodes, the average number of selenoproteins was computed, and a white dot indicates that all species contain EF-Sec genes. The phylogeny was obtained from NCBI taxonomy. (TIF) [file pcbi.1005383.s008.tif]

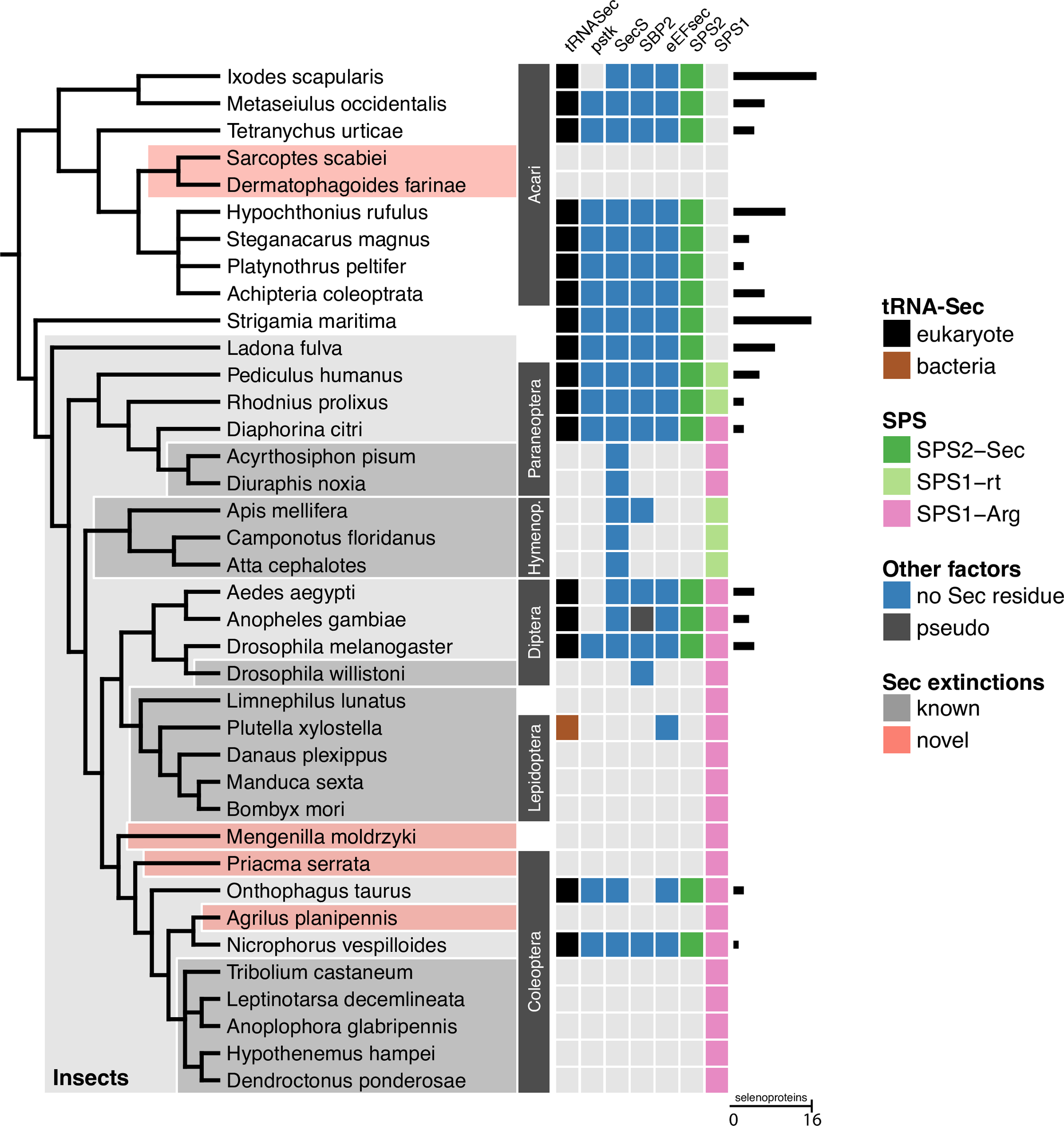

Supplement: S9 Fig — Species tree including a subset of the arthropod genomes analyzed in this work. The shaded boxes indicate known (dark grey) and novel (red) Sec extinctions. Each species is annotated with the presence of tRNASec, the protein factors of the Sec machinery (including the selenoprotein SPS2-Sec), and SPS1 genes. SPS1-Arg corresponds to SPS genes with an arginine codon at the homologous Sec position, and SPS1-rt corresponds to SPS genes with a UGA codon, in which a readthrough event occurs but the inserted amino acid is not known (see [6]). The black horizontal bar indicates the number of selenoproteins. The topology of the Coleoptera lineage was adapted according to [67]. (TIF) [file pcbi.1005383.s009.tif]
