# Supplementary material for: Computational identification of the selenocysteine tRNA (tRNASec) in genomes
Source: PLoS Comput Biol. 2017 Feb 13;13(2):e1005383. doi: 10.1371/journal.pcbi.1005383 (PMC5330540; doi:10.1371/journal.pcbi.1005383)
Supplement: S1 Text — (PDF) [file pcbi.1005383.s011.pdf]

Secmarker

S1 Text: Supplementary materials

1. Benchmark of tRNA-Sec prediction methods 2
2. Benchmark with tRNAscan-SE 2.0 4
3. Benchmark with RF01852 5
4. Examples of false positive tRNA-Sec predictions in the benchmark set. 6
5. Phytophthora capsici tRNA-Sec. 7
6. Sec-containing Thioredoxin reductase (TR) in Phytophthora ramorum. 8
7. Sec trait in Burkholderia. 9
8. Non-G73 top scoring predictions in bacteria 11
9. References 12

1. Benchmark of tRNA-Sec prediction methods

A) Benchmark in eukaryotes
A total of 212 eukaryotic genomes (105 with Sec, and 107 without Sec) were analyzed. Secmarker predicted tRNA-Sec in 104 genomes from the positive set (105), achieving a sensitivity of 99%, compared to 94% and 61% for aragorn and tRNAscan-SE, respectively. There was a single Sec-containing genome without Secmarker predictions (false negative), that of the protist *Phytophthora capsici* (Fig 2). Like Secmarker, tRNAscan-SE did not predict any tRNA-Sec candidate. However, aragorn predicted two tRNA-Sec genes in the genome of *P. capsici*. Manual inspection of the aragorn candidates revealed that these did not fit the eukaryotic tRNA-Sec model (section 2 in this file). We concluded that the tRNA-Sec predictions by aragorn in the genome of *P. capsici* were actually false positives. Notwithstanding, since the rest of Sec machinery and a Sec-containing thioredoxin reductase gene were found in the genome [1], we expected tRNA-Sec to be present as well, but most likely missing from the genome assembly. We thus analyzed raw sequencing data available from *P. capsici* with Secmarker. We identified a tRNA-Sec supported by several genomic reads, containing the full sequence of the gene (section 3 in this file). This confirmed the presence of tRNA-Sec in *P. capsici*. Therefore, despite this genome was counted as a Secmarker false negative, this was due to incompleteness of the genome assembly available, rather than lack of accuracy of the program.

The specificity of Secmarker in eukaryotic genomes was 99%, compared to 62% and 49% for tRNAscan-SE and aragorn, respectively. Unlike Secmarker, the other two programs produced numerous false positive predictions in fungi and land plants (Fig 2A), both known to lack selenoproteins [2]. The only false positive by Secmarker was a bona fide prediction in the genome of *Phytophthora ramorum*. The presence of a good tRNA-Sec candidate for *P. ramorum* is in apparent contradiction with the the lack of selenoprotein genes and other Sec machinery factors in the genome of this species, as reported in [1]. Suspiciously, the other three genomes available from the genus *Phytophthora*, in contrast, exhibit the complete Sec machinery and at least one bona fide selenoprotein gene. Although selenoprotein extinctions have been reported in some species within otherwise Sec-containing genus [3], this could also reveal that the genome assembly analyzed in [1] was incomplete. Thus, we analyzed a more recent, more complete assembly, and found all the markers of Sec utilization, as well as a Sec-containing thioredoxin reductase gene with a SECIS element (section 4 in this file). We concluded, therefore, that *P. ramorum* was wrongly predicted to lack Sec in [1]; this species actually utilizes Sec and Secmarker and aragorn correctly identified the tRNA-Sec gene.

We investigated the overlap between the tRNA-Sec predictions by the different programs. Secmarker showed the highest fraction of predictions with support from at least another program (83.0%) compared to aragorn and tRNAscan-SE (39.7% and 30.6%, respectively).

There were 35 predictions by Secmarker in Sec-containing genomes that did not overlap any prediction from the other two programs (Fig 2B). Only eight of them corresponded to the top scoring prediction in the genome. Such predictions were in six protists and in *Daphnia pulex* (discussed in the main text). The top scoring prediction in *Monodelphis domestica* was among the 35, but it showed pseudogene features. The remaining corresponded to genomes with multiple predictions, and the top scoring one was not among the 35. Some of them were likely to be Secmarker false positive predictions.

B) Benchmark in bacteria.
A total of 217 bacterial genomes were included in the test, with 42 of them (19%) predicted to utilize Sec. Secmarker and aragorn achieved perfect sensitivity (100%), while tRNAscan-SE

missed tRNA-Sec in six Sec-containing bacterial genomes (sensitivity 86%). Specificity was higher for Secmarker (99%) than for aragorn (87%) and tRNAscan-SE (92%). Secmarker produced two false positives, both in the genus *Burkholderia*. The sequences of these two tRNA-Sec candidates are identical. Both of them were predicted also by aragorn, and missed by tRNAscan-SE. These two genomes were predicted to lack Sec due to the absence of the gene *selD* in the genome assembly [1]. However, we were able to identify the rest of the Sec machinery, as well as a Sec-containing FDH gene (section 5 this file). This supports that, although these two genomes appeared as false positives in our benchmark, Sec is actually used by these organisms and Secmarker and aragorn correctly identified their tRNA-Sec gene.
There was a good overlap between the predictions produced by the three programs in bacterial genomes (Fig 3B). All but one of the tRNA-Sec predictions in Sec-containing genomes were predicted by at least two programs, and 37 out of 48 were predicted by the three of them.

C) Benchmark in archaea.
Since archaeal genomes were scarcely represented in [1], we analyzed a larger number of genomes in this kingdom (Fig 4). We used the presence of the genes *selD* and *EF-Sec* identified by Selenoprofiles [4] to predict Sec utilization in a total of 213 archaeal genomes. We predicted 14 such genomes (7%) to use Sec. Secmarker obtained perfect sensitivity (100%), while aragorn sensitivity was 93% and tRNAscan-SE sensitivity was 57%, with one and six false negatives, respectively (Fig 4). RF01852 sensitivity, ran with the recommended parameters (http://rfam.xfam.org/family/tRNA-Sec#tabview=tab9), was 92.9%, missing the tRNA-Sec in *Lokiarchaeota*. Perfect specificity was reached with Secmarker and tRNAscan-SE (100%), while aragorn obtained a specificity of 98%, with five false positives. Manual inspection of the false positives by aragorn revealed that their sequences did not fit the the archaeal tRNA-Sec model (section 2 in this document). With this, and the lack of other Sec machinery factors and selenoproteins in these genomes, we concluded that they are indeed false positives. In addition, we noticed that aragorn predicted a second tRNA-Sec in the genome of *Methanococcus voltae A3*. This second tRNA had no variable arm, therefore it was a clear false positive (section 2 in this document). That same tRNA was the only candidate predicted by tRNAscan-SE in the genome of *M. voltae A3*. tRNAscan-SE, therefore, missed the real tRNA-Sec in this genome, even though in our benchmark it was considered a true positive prediction.

2. Benchmark with tRNAscan-SE 2.0

The benchmark of Secmarker included tRNAscan-SE v1.23. A newer version 2.0 is available through a web server (http://trna.ucsc.edu/tRNAscan-SE/). However, the program is not yet available for download (as of 11/2016). Due to limitation in the maximum input size, we cannot replicate the benchmark (641 genomes) through the web server. We thus decided to perform a more limited test, running tRNAscan-SE 2.0 with a small set of false positives and true positives obtained from our benchmark set. We retrieved the 102 SeC predictions obtained with tRNAscan-SE v1.23 in selenoproteinless genomes (considered here as false positives), and the 159 Secmarker top scoring predictions from selenoprotein-containing genomes (considered here as true positives). All predictions were extended 25 bases at both sides. The “sequence source” option in the tRNAscan-SE 2.0 web server was set as “Bacterial”,”Archaeal” or “Eukaryotic”, according to the source organism. The rest of parameters were set to default values.
The results with the newer tRNAscan-SE 2.0 did improve compared to version 1.23, specially in sensitivity (S1.1 Table). All but one of the prokaryotic true positives (TP) were identified, and the number of detected TP in eukaryotes more than doubled. Yet, Secmarker still appears to perform much better. tRNAscan-SE 2.0 missed 14 eukaryotic TP. Regarding the false positives (FP), the eukaryotic ones were reduced to 54, but the 14 prokaryotic were mispredicted again (13 of them belong to *Mycoplasma*, with a variation in the genetic code where UGA encodes for Trp).

3. Benchmark with RF01852

The Rfam database provides a CM for tRNA-Sec (RF01852). In order to benchmark Secmarker (which includes three manually curated CM models) against the single RF01852 model, we downloaded the RF01852 and ran it on our set of genomes with cmsearch (Infernal v 1.1.1). The parameters recommended in the curation page (http://rfam.xfam.org/family/RF01852#tabview=tab9) were used: 'cmsearch --nohmmonly -T 25.39'. The results were then parsed to exclude those hits with score lower than 47.0 ("gathering threshold").

The results were compared with Secmarker. RF01852 obtained a higher number of predictions, 5,133 vs 3,421. The overlap of the two methods was 3,293 (63%) from a total of 5,261 (the union of the two runs); 128 (2%) were predicted only by Secmarker, and 1,840 (35%) were predicted only by RF01852. We further analyzed all 5261 by running cmsearch with RF00005 (canonical tRNA). 98% of the results predicted only by RF01852 had a higher score when aligned to RF00005, suggesting they are in fact canonical tRNAs. That proportion was much lower for the results predicted only by Secmarker, 7%. Among the overlapping predictions, only 1% had a higher score with RF00005.

Besides having better performance, Secmarker has the advantage of assigning the domain (bacteria, archaea or eukaryota).

4. Examples of false positive tRNA-Sec predictions in the benchmark set.

Eight false positive predictions (A-H) from the genomes in the benchmark set returned by aragorn. Each prediction is described by five lines corresponding to: the species name, the sequence identifier (preceded by ">"), the tabular output with the positions between brackets (preceded by a "c" for complementary strand), the sequence of the prediction, and finally the predicted secondary structure. The lower case letters in the secondary structure indicate the position of the loops in the different arms: "d" for D arm, "v" for variable arm, and "t" for T arm. "AAA" indicates the anticodon. None of the following predictions fit the tRNA-Sec structure.

A) Species**: *Phytophthora capsici***
>PHYCAscaffold_2
3 tRNA-seC c[1712035,1712126] 37 (tca)
```
gatctcgtggtcgatcgcgctccccgactaggatcttcagtgtcctgaagagatcctgaattgctacctggatcgcgtccaggcccagatca
((((.(. (((dddddddddd))))((((((  AAA  )))))) ((.(((vvvvv))).)) ((((ttttttt))))).).)))))
```

B) Species**: *Phytophthora capsici***
>PHYCAscaffold_35
1 tRNA-seC [68443,68519] 38 (tca)
```
gtgtgcgacttggcgggttcgtctaggctgcgtggcttcagatcacccagcgcgggttcgagccccgcctgcgtgtg
((((((.   (.(ddddddddddd).) (.((((  AAA  )))).)   (((((ttttttt))))).)))))))
```

C) Species**: *Sulfolobus solfataricus 98/2***
>ACUK01000205.1 Sulfolobus solfataricus 98/2 contig208, whole genome shotgun sequence
1 tRNA-seC c[4419,4512] 35 (tca)
```
gccgccgtagctcagcctggttagagcgccggactcatacgcgtaatatccgggaaatccggttgtccggggttcaaatccccgcggcggcacc
((((((  ((((dddddddddd))))((..(  AAA  )..))     ((((vvvvv))))     (((((ttttttt)))))))))))
```

D) Species**: *Sulfolobus islandicus HVE10/4***
>CP002426.1 Sulfolobus islandicus HVE10/4, complete genome
16 tRNA-seC [1522796,1522889] 35 (tca)
```
gccgccgtagctcagcctggttagagcgccggactcatacgcgtaatatctgggaaatccggttgtccggggttcaaatccccgcggcggcacc
((((((  ((((dddddddddd))))((..(  AAA  )..))     ((((vvvvv))))     (((((ttttttt)))))))))))
```

E) Species**: *Halococcus hamelinensis 100A6***
>AOMB01000020.1 Halococcus hamelinensis 100A6 contig_20, whole genome shotgun sequence
1 tRNA-seC c[38222,38312] 32 (tca)
```
gccatcgttcgaccgcgggtcggcggcggcttcaggctgtcgtcggtttcgccgagagcgagcgcgagggatcgtggtcctcgcgcgggta
(((..(((  ((((ddd))))  ((((((  AAA  ))))))     (((((vvvvv)))))   (((((ttttttt))))))))..)))
```

F) Species**: *Halococcus sp. 197A***
>BAFM01000011.1 Halococcus sp. 197A DNA, contig: Hcc11, whole genome shotgun sequence
1 tRNA-seC c[29372,29467] 37 (tca)
```
tttggcgttcgagcaggggatgtggctcgtcgcgtttcacctgcgatttgagttcggcagcagtgtcgagcaccgtttcgagttcgtcggccgaga
(((((.   ((((dddddddddd)))) (((((  AAA  )))))      (((((((vvvv))))))) ..((.ttttttt.))...))))))
```

G) Species**: *Haloplanus natans DSM 17983***
>KE386573.1 Haloplanus natans DSM 17983 genomic scaffold HALNADRAFT_scaffold1.1, whole genome shotgun sequence
34 tRNA-seC [2075394,2075489] 36 (tca)
```
gtgttcgaatggggcgcgtggacggccacgagtcgtcacgaccggaggaccgtctcgccggtgggtcttcgactgttcggttcggttcccaacata
((((.(.   .(((ddddddddd))).(..((  AAA  ))..)(((.(((((vvvvv))))).)))  (((((ttttttt))))).).)))))
```

H) Species**: *Methanococcus voltae A3***
>CP002057.1 Methanococcus voltae A3, complete genome
10 tRNA-seC c[228918,228991] 35 (tca)
```
gtcaaggtagtctagtccggccaggcaacggacatcagattcgtgcaacaggggttcaaatcccttccttggct
((((((  ((((ddddddddd)))) (((((  AAA  )))))     (((((ttttttt))))))))))))
```

5. *Phytophthora capsici* tRNA-Sec.

A) Secmarker results in genomic reads.

Analysis of *P. capsici* raw genomic reads with Secmarker revealed a tRNA-Sec candidate. Two different samples were used (SRR943799 and SRR945695). The read identifier and positions of the tRNA are included in the header of each sequence.

```
>SRR943799.fasta.etRNAsec.3 tRNA-Sec(e) chromosome:SRR943799.568178 strand:- positions:165-251
target:SRR943799.fasta infernal_score:83.1 truncated:False discr_base:G anticodon:UCA
GCAGGGGUGAUCUUGGCUGGUGCUGAGUGCAGACUUCAAAUCUGUAGGCCAUUCGUUUGGCAGGAGUUCGAUUCUCCCUCGCUUGCG
>SRR943799.fasta.etRNAsec.2 tRNA-Sec(e) chromosome:SRR943799.262468 strand:- positions:109-195
target:SRR943799.fasta infernal_score:83.1 truncated:False discr_base:G anticodon:UCA
GCAGGGGUGAUCUUGGCUGGUGCUGAGUGCAGACUUCAAAUCUGUAGGCCAUUCGUUUGGCAGGAGUUCGAUUCUCCCUCGCUUGCG
>SRR943799.fasta.etRNAsec.1 tRNA-Sec(e) chromosome:SRR943799.84635 strand:- positions:517-603
target:SRR943799.fasta infernal_score:83.1 truncated:False discr_base:G anticodon:UCA
GCAGGGGUGAUCUUGGCUGGUGCUGAGUGCAGACUUCAAAUCUGUAGGCCAUUCGUUUGGCAGGAGUUCGAUUCUCCCUCGCUUGCG
>SRR945695.fasta.etRNAsec.3 tRNA-Sec(e) chromosome:SRR945695.19108665 strand:- positions:105-191
target:SRR945695.fasta infernal_score:83.1 truncated:False discr_base:G anticodon:UCA
GCAGGGGUGAUCUUGGCUGGUGCUGAGUGCAGACUUCAAAUCUGUAGGCCAUUCGUUUGGCAGGAGUUCGAUUCUCCCUCGCUUGCG
>SRR945695.fasta.etRNAsec.2 tRNA-Sec(e) chromosome:SRR945695.14526540 strand:- positions:113-199
target:SRR945695.fasta infernal_score:83.1 truncated:False discr_base:G anticodon:UCA
GCAGGGGUGAUCUUGGCUGGUGCUGAGUGCAGACUUCAAAUCUGUAGGCCAUUCGUUUGGCAGGAGUUCGAUUCUCCCUCGCUUGCG
>SRR945695.fasta.etRNAsec.1 tRNA-Sec(e) chromosome:SRR945695.2975118 strand:+ positions:11-97
target:SRR945695.fasta infernal_score:83.1 truncated:False discr_base:G anticodon:UCA
GCAGGGGUGAUCUUGGCUGGUGCUGAGUGCAGACUUCAAAUCUGUAGGCCAUUCGUUUGGCAGGAGUUCGAUUCUCCCUCGCUUGCG
```

B) Cloverleaf secondary structure of *P. capsici* tRNA-Sec.

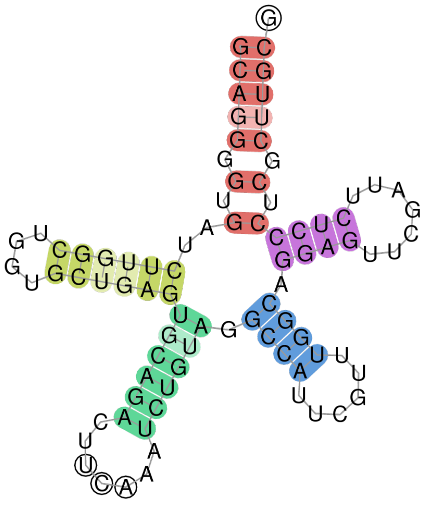

6. Sec-containing Thioredoxin reductase (TR) in *Phytophthora ramorum.*

A) Predicted aminoacid sequence of the *P. ramorum* TR. Sec (U) is found in the penultimate residue.

```
>TR.1.selenocysteine chromosome:gi|169255255|gb|DS566053.1| strand:+
positions:158827-160371
GTADALAAVEEEAEIPSADAEEETSYDYDLVVIGGGSGGLACSKEAASFGKKVCVLDYVK
PSPQGTSWGLGGTCVNVGCIPKKLMHQSSLIGEVMHHDSANFGWNVASEGSAPTFNWKQL
VSNVDAYIKSINFKYKVELRSKYVKYENFLGSFVDPHTLELWHHRKGTKQITTRDVVIAV
GGRPKELSCPGGEHAISSDDIFWMKKKEPGKTLVVGASYVALECAGFLKGMGYEVKVMVR
SILLRGFDQDMANKIGEYMEVQSGIEFIRKTVPQSITTLENGQLLVKWTNEDGESCEEAF
DTVLNATGRDPDVAKLGLDKAGVKLNEKSGRIWVKNEQTSTSNIYALGDVIDAPELTPVA
IQAGRLLSRRLYNNSTAQMDYEKVCTAVFTPIEYGCCGLSEEDSKERFGEDNVEVYHTNF
VPLEWSLSETRTSAESCYVKVICDKTRDKFVVGFHYLGPNAGEVTQAMGLAMKLGFTYDQ
MVDTVGIHPTTAESFTTLEVTKSSGGATTGGGCUG
```

B) *Bona fide* SECIS element prediction in *P. ramorum* TR.

SECIS predictions obtained with Seblastian [5].

```
-SECIS: TR.1.selenocysteine.secis1     grade:A
 Chromosome:gi|169255255|gb|DS566053.1| Strand:+
 Positions:160565-160637     CDS-Distance:193     Sec-Distance:196
 Found by:  Infernal (score=24.83) Covels (score=21.75)
 Free Energy: -20.1
 Type: 2

         ..(((((((.......(((((((((((((((...............)))))))..))))))))........)))))))..
 model   uucggucaugcgUuaAUGAcGgccuggcccuAAAcCcuuuuuggggcgggcca--ggcCuGAUGuuuuuucaugaccggc
         .({(((((((--....([[{(((((((((.....(((.....)))...)))))..))))}]])----..-)))))))}).
 target  CUAGCGCUGA--GUAAUGACGCUCUUUCCAGAAAUCCCUAGCGGGAGGGGAAAUCGAGCUGAG----AA-UCGGCGCAGC
                         ****           ^^^                         ****
```

7. Sec trait in *Burkholderia*.

Two genomes from the benchmark set, belonging to the *Burkholderia* genus, were considered false positives, because they were classified as selenoproteinless in [1] due to the absence of the gene *selD* in their genome sequences. However, we were able to identify the rest of the Sec machinery genes, and a Sec-containing FDH gene. In the two genomes, the genes *tRNA-Sec, selB*, *selA* and *FDH* were found adjacent to each other in the same strand, very likely belonging to the same operon.

A) *Burkholderia pseudomallei K96243* tRNA-Sec, SelB, SelA and FDH predictions.

```
>Burkholderia_pseudomallei_K96243.btRNAsec.1 tRNA-Sec(b) chromosome:gi|53721039|ref|
NC_006351.1| strand:- positions:2282352-2282444 target:Burkholderia_pseudomallei_K96243/
genome.fa infernal_score:87.5 truncated:False discr_base:G
GGAAGGCAUUCGUAUCCGGUGGUGCGGCUGGGCUUCAAACCCAGUUGGUGCGGUCAGCCCGCGCCAGGUCGGUUCGACUCCGGCUGCCU
UCCG

>selB.1.homologue chromosome:gi|53721039|ref|NC_006351.1| strand:- positions:
2282555-2284477 target:Burkholderia_pseudomallei_K96243/genome.fa
prediction_program:blast
MIVGTAGHIDHGKTTLVRALTGVDTDRLKEEKARGISIELGYAYTPLPNGDVLGFIDVPG
HEKLVHTMAAGACGIDFALVVIAADDGVMPQTREHLSILQLLGVAHGALALTKCDRVDAA
RVARVRDEIRAWLAASPLADAPVFETRASEPDDAGVAALNAHLRDTALAWRARRDDGLFR
LAVDRVFTLAGQGTVVTGTAVAGRVRTGDSLAVARTGETVRVRSIHAQNRATDVGHAGER
CALNLAGIDKAALARGDAIVDARLATLSPRIDVELTLTADADLTITHWTPLHVHLGTLHR
VAHVALLEGETLGPGRRARAQLNFTEPVFAAPGDRFIVRDAQATRTVGGGRVLDPFGPAR
KRRTRARRAWLDALAAWLDEGRLDALLDEAPLGIARATLMHLTGLPAQAWALPADAVSVA
APGKHADEARVLARGHWDALRTRVLDALAAFHQRSPDEQGPDVARLRRIAAPLADDALWR
ALTDALIAEGAIVRSGPWLHLPSHAVSFDAAEEALAGRLLPLVAAGRYDPPWVRDHAAAT
LMAEDAVRALLRKLARRGDVHQVVRDLFYHRDVIAELARLIARLAGEHGGGLDAATFRDA
TGLGRKRAIQILEFFDRVGYTRFHRDLHWLRADSRLLAGSR

>selA.1.homologue chromosome:gi|53721039|ref|NC_006351.1| strand:- positions:
2284477-2285880 target:Burkholderia_pseudomallei_K96243/genome.fa
prediction_program:blast
LARAPSVERVLSSEEMQPLVDEYGRTRALAAVRASLAAWRDAARRDPAAAGTPDDARIAA
DVRARLAADAAAAMRAVFNLTGTVLHTNLGRALLPDDAVRAVVQALTQPMNLEFDLATGR
RGDRDDLIGALACELTGAEAATVVNNNAAAVLLVLSALAPRREVVVSRGELVEIGGAFRI
PDIMSRAGAKLREVGTTNRTHLADYADAIGPRTALLMKVHCSNYAISGFTKEVALRELAP
LARERGVPVAVDLGSGTLVDLTRWGLPKETTVRETVEAGADLVTFSGDKLLGGPQAGLIV
GRRDLIAKIKKHPLKRALRVGKLTLAALEPVLRLYQTPEFLRERLTTLRLLTRAQADIAA
TAERVRPALQRALGAAFAVDVEPMFSQIGSGALPVDQLPSYGLVVRASGGKRRGRALAQL
DAHLRGWPRPVLGRIADDALRLDLRCLEAGDEAAFIAQCAQAPTGPRA

>fdha.20.selenocysteine chromosome:gi|53721039|ref|NC_006351.1| strand:- positions:
2288607-2291693 target:Burkholderia_pseudomallei_K96243/genome.fa
prediction_program:blast
NLPETTMLQLSRRQFLKLSATTLAGSSLALMGFSPAEALAEVRQYKLARTVETRNTCPYC
SVGCGILMYGLGDGAKNATSSIVHIEGDPDHPVNRGTLCPKGASLIDFIHSPSRLTQPEY
RAAGSDKWQPISWSDALDRIAKLMKADRDANFVETTDDGMKVNRWLTTGMLAASAGSNEV
GYLTHKTVRSMGMLAFDNQARVUHGPTVAGLAPTFGRGAMTNHWVDIKNADVILVMGGNA
AEAHPCGFKWVTEAKAHRNARLVVVDPRFTRTASVADYYAPIRTGTDIAFLGGVIHYLLT
NDKIQHEYVKHYTDFSFIVREDFAFDDGIYSGYDADKHAYPDKSTWDYERGDDGFVKVDE
TLAHPRCVYNLLKQHYARYTPEMVEKICGTPKDKFLKVCEMLATTAVPGRAGTVLYALGW
THHSVGAQMIRTGAMVQLLLGNIGIAGGGMNALRGHSNIQGLTDLGLMSNLLPGYMTLPM
QAEQDFDAYIQKRAQQPLRPNQLSYWKNYRAFHVSFMKAWWGDAASAENNWGYDYLPKLD
KQYDLLQTIELMHAGKMNGYICQGFNPLAAAPSKRKTSEALAKLKWLVIMDPLATETSEF
WKNHGEFNDVDSSKIQTEVFRLPTSCFAEERGSLVNSGRVLQWHWQGAEPPGQAKSDLEI
MSGIFLRMRDMYRKDGGKYPDPIVNLSWPYANPESPTPEELAMEFNGRALADLPDPKDPT
KTLVKKGEQLAGFAQLKDDGTTASGCWIFCGAWTQAGNQMARRDNADPTGIGQTLNWAWA
WPANRRILYNRASCDVNGKPFDPSRKLIGWNGKTWTGADVPDYKLDEPPETGMGPFIMNP
EGVARFFARAGMNEGPFPEHYEPFETPLAANPLHPGNPRALNNPAARVFPDDRASFGKVD
QFPHVATTYRLTEHFHYWTKHARLNAIVQPQQFVEIGEDLAKEIGVAHGEQVKVSSNRGH
IVAVALVTKRIKPLMVDGRKVQTVGVPLHWGFKGLTKPGYLANTLTPSVGDGNSQTPEFK
SFLVKVEKA
```

B) *Burkholderia mallei ATCC 23344* tRNA-Sec, SelB, SelA and FDH predictions.
FDH in this genome has an in-frame stop codon (TAG), indicated with "*" in the sequence below (fdha.17.pseudo). It could be due to pseudogenization of the gene, although we can not discard a sequencing error in the genome assembly.

```
>Burkholderia_mallei_ATCC_23344.btRNAsec.1 tRNA-Sec(b) chromosome:gi|77358719|
ref|NC_006349.2| strand:- positions:1830021-1830113
target:Burkholderia_mallei_ATCC_23344/genome.fa infernal_score:87.5
truncated:False discr_base:G
GGAAGGCAUUCGUAUCCGGUGGUGCGGCUGGGCUUCAAACCCAGUUGGUGCGGUCAGCCCGCGCCAGGUCGGUUCGACUC
CGGCUGCCUUCCG

>selB.1.homologue chromosome:gi|77358719|ref|NC_006349.2| strand:- positions:
1830224-1832146 target:Burkholderia_mallei_ATCC_23344/genome.fa
prediction_program:blast
MIVGTAGHIDHGKTTLVRALTGVDTDRLKEEKARGISIELGYAYTPLPNGDVLGFIDVPG
HEKLVHTMAAGACGIDFALVVIAADDGVMPQTREHLSILQLLGVAHGALALTKCDRVDAA
RVARVRDEIRAWLAASPLADAPVFETRASEPDDAGVAALNAHLRDTALAWRARRDDGLFR
LAVDRVFTLAGQGTVVTGTAVAGRVRTGDSLAVARTGETVRVRSIHAQNRATDVGHAGER
CALNLAGIDKAALARGDAIVDARLATLSPRIDVELTLTADADLTISHWTPLHVHLGTLHR
VAHVALLEGETLGPGRRARAQLNFTEPVFAAPGDRFIVRDAQATRTVGGGRVLDPFGPAR
KRRTRARRAWLDALAAWLDEGRLDALLDEAPLGIARATLMHLTGLPAQAWALPADAVSVA
APGKHADEARVLARGHWDALRTRVLDALAAFHQRSPDEQGPDVARLRRIAAPLADDALWR
ALTDALIAEGAIVRSGPWLHLPSHAVSFDAAEEALAGRLLPLVAAGRYDPPWVRDHAAAT
LMAEDAVRALLRKLARRGDVHQVVRDLFYHRDVIAELARLIARLAGEHGGGLDAATFRDA
TGLGRKRAIQILEFFDRVGYTRFHRDLHWLRADSRLLAGSR

>selA.1.homologue chromosome:gi|77358719|ref|NC_006349.2| strand:- positions:
1832146-1833549 target:Burkholderia_mallei_ATCC_23344/genome.fa
prediction_program:blast
LARAPSVERVLSSEEMQPLVDEYGRTRALAAVRASLAAWRDAARRDPAAAGTPDDARIAA
DVRARLAADAAAAMRAVFNLTGTVLHTNLGRALLPDDAVRAVVQALTQPMNLEFDLATGR
RGDRDDLIGALACELTGAEAATVVNNNAAAVLLVLSALAPRREVVVSRGELVEIGGAFRI
PDIMSRAGAKLREVGTTNRTHLADYADAIGPRTALLMKVHCSNYAISGFTKEVALRELAP
LARERGVPVAVDLGSGTLVDLTRWGLPKETTVRETVEAGADLVTFSGDKLLGGPQAGLIV
GRRDLIAKIKKHPLKRALRVGKLTLAALEPVLRLYQTPEFLRERLTTLRLLTRAQADIAA
TAERVRPALQRALGAAFAVDVEPMFSQIGSGALPVDQLPSYGLVVRASGGKRRGRALAQL
DAHLRGWPRPVLGRIADDALRLDLRCLEAGDEAAFIAQCAQAPTGPRA

>fdha.17.pseudo chromosome:gi|77358719|ref|NC_006349.2| strand:- positions:
1836276-1839362 target:Burkholderia_mallei_ATCC_23344/genome.fa
prediction_program:blast
NLPETTMLQLSRRQFLKLSATTLAGSSLALMGFSPAEALAEVRQYKLARTVETRNTCPYC
SVGCGILMYGLGDGAKNATSSIVHIEGDPDHPVNRGTLCPKGASLIDFIHSPSRLTQPEY
RAAGSDKWQPISWSDALDRIAKLMKADRDANFVETTDDGMKVNRWLTTGMLAASAGSNEV
GYLTHKTVRSMGMLAFDNQARVUHGPTVAGLAPTFGRGAMTNHWVDIKNADVILVMGGNA
AEAHPCGFKWVTEAKAHRNARLVVVDPRFTRTASVADYYAPIRTGTDIAFLGGVIHYLLT
NDKIQHEYVKHYTDFSFIVREDFAFDDGIYSGYDADKHAYPDKSTWDYERGDDGFVKVDE
TLAHPRCVYNLLKQHYARYTPEMVEKICGTPKDKFLKVCEMLATTAVPGRAGTVLYALGW
THHSVGAQMIRTGAMVQLLLGNIGIAGGGMNALRGHSNIQGLTDLGLMSNLLPGYMTLPM
QAEQDFDAYIQKRAQQPLRPNQLSYWKNYRAFHVSFMKAWWGDAASAENNWGYDYLPKLD
KQYDLL*TIELMHAGKMNGYICQGFNPLAAAPSKRKTSEALAKLKWLVIMDPLATETSEF
WKNHGEFNDVDSSKIQTEVFRLPTSCFAEERGSLVNSGRVLQWHWQGAEPPGQAKSDLEI
MSGIFLRMRDMYRKDGGKYPDPIVNLSWPYANPESPTPEELAMEFNGRALADLPDPKDPT
KTLVKKGEQLAGFAQLKDDGTTASGCWIFCGAWTQAGNQMARRDNADPTGIGQTLNWAWA
WPANRRILYNRASCDVNGKPFDPSRKLIGWSGKTWTGADVPDYKLDEPPETGMGPFIMNP
EGVARFFARAGMNEGPFPEHYEPFETPLAANPLHPGNPRALNNPAARVFPDDRASFGKVD
QFPHVATTYRLTEHFHYWTKHARLNAIVQPQQFVEIGEDLAKEIGVAHGEQVKVSSNRGH
IVAVALVTKRIKPLMVDGRKVQTVGVPLHWGFKGLTKPGYLANTLTPSIGDGNSQTPEFK
SFLVKVEKA
```

8. Non-G73 top scoring predictions in bacteria

The following predictions were the top scoring in the corresponding genomes, but they lacked the residue G73. We could identify *selA* and *selB* genes in the genome of *Aggregatibacter actinomycetemcomitans* and in the two genomes from the genus *Sulfurimonas.* The two *Sulfurimonas* predictions include the CCA terminal tail.

```
>Aggregatibacter_actinomycetemcomitans_D11S-1.btRNAsec.1 tRNA-Sec(b)
chromosome:NC_013416.1 strand:+ positions:322540-322631
target:Aggregatibacter_actinomycetemcomitans_D11S-1/genome.fa infernal_score:
73.5 truncated:False discr_base:C
GGAAGAUCGUCGUUUCCGGUGAGGCGGCAGGACUUCAAAUCCUGUUGAGGCUGCCAGCAGUCUCGGGUAGGUUCAACUCC
UACGAUCGCCAC

>Sulfurimonas_denitrificans_DSM_1251.btRNAsec.1 tRNA-Sec(b) chromosome:gi|
78776201|ref|NC_007575.1| strand:- positions:862215-862314
target:Sulfurimonas_denitrificans_DSM_1251/genome.fa infernal_score:57.1
truncated:False discr_base:A
UGGGGGGCAUGUGAUUCUGGUGAACACCGCAGACUUCAAACCUGAUUGGAGGUUUUGCUGACAAAACCUCGGGAGGUUCG
AUUCCUUCGCUCUCUCACCA

>Sulfurimonas_gotlandica_GD1.btRNAsec.1 tRNA-Sec(b) chromosome:AFRZ01000001.1
strand:+ positions:105325-105424 target:Sulfurimonas_gotlandica_GD1/genome.fa
infernal_score:53.6 truncated:False discr_base:A
UGGGGGGCAUGUGACCCUGGUGGGCACCACAGGUUUCAAACCUGAUUGGAGGUUUUUGUGUAAACGCCUCGGGGGGUUCG
AUUCCUUCGCCUUCUCACCA


>Bacillus_licheniformis_WX-02.btRNAsec.1 tRNA-Sec(b) chromosome:JH636050.1
strand:+ positions:1111213-1111300 target:Bacillus_licheniformis_WX-02/genome.fa
infernal_score:43.9 truncated:False discr_base:C
UGCCGGGGUGGUGGAAUUGGCAGACACACAGGACUUCAAAUCCUGCGGUAGGUGACUACCGUGCCGGUUCAAGUCCGGCC
CUCGGCAC

>Capnocytophaga_canimorsus_Cc5.btRNAsec.2 tRNA-Sec(b) chromosome:NC_015846.1
strand:- positions:141468-141560 target:Capnocytophaga_canimorsus_Cc5/genome.fa
infernal_score:44.1 truncated:False discr_base:C
CGCAACGUUGGUGUAAGGGUAGCACACAAGCCUUCAAAGCUUGGUAGCGUAUCUCCAGUACGCAGGAGUGGGUUCGAGUC
CCAUACGUUGCUC
```

9. References

1. Mariotti M, Santesmasses D, Capella-Gutierrez S, Mateo A, Arnan C, Johnson R, D'Aniello S, Yim SH, Gladyshev VN, Serras F, Corominas M, Gabaldón T, & Guigó R. (2015). Evolution of selenophosphate synthetases: emergence and relocation of function through independent duplications and recurrent subfunctionalization. Genome Research, 25(9), 1256–67. doi:10.1101/gr.190538.115
2. Lobanov, A. V, Hatfield, D. L., & Gladyshev, V. N. (2009). Eukaryotic selenoproteins and selenoproteomes. Biochimica et Biophysica Acta, 1790(11), 1424–8. doi:10.1016/j.bbagen.2009.05.014
3. Chapple, C. E., & Guigó, R. (2008). Relaxation of selective constraints causes independent selenoprotein extinction in insect genomes. PloS One, 3(8), e2968. doi:10.1371/journal.pone.0002968
4. Mariotti, M., & Guigó, R. (2010). Selenoprofiles: profile-based scanning of eukaryotic genome sequences for selenoprotein genes. Bioinformatics (Oxford, England), 26(21), 2656–63. doi:10.1093/bioinformatics/btq516
5. Mariotti, M., Lobanov, A. V, Guigo, R., & Gladyshev, V. N. (2013). SECISearch3 and Seblastian: new tools for prediction of SECIS elements and selenoproteins. Nucleic Acids Research, 41(15), e149. http://doi.org/10.1093/nar/gkt550
